# Strain accumulation and release associated with the occurrence of SSEs in the subduction zones of the Japanese Islands

**DOI:** 10.1038/s41598-023-28016-1

**Published:** 2023-01-25

**Authors:** Hiroki Kawabata, Shoichi Yoshioka

**Affiliations:** 1 Department of Planetology, Graduate School of Science, Kobe University, Rokkodai-cho 1-1, Nada Ward, Kobe, 657-8501 Japan; 2 Present Address: Research Center for Urban Safety and Security, Kobe University, Rokkodai-cho 1-1, Nada Ward, Kobe, 657-8501 Japan

**Keywords:** Solid Earth sciences, Geodynamics, Seismology

## Abstract

We investigated the spatiotemporal changes in strain associated with the occurrence of slow slip events (SSEs) in the subduction zones of the Japanese Islands and compared the spatial distribution of both the amount of strain accumulated for the period before and during the SSEs release using time series data from the Global Navigation Satellite System (GNSS). In this study, four SSEs were analysed: the Tokai long-term SSE (2000–2005), the Boso-Oki short-term SSE (2007), and the Bungo Channel long-term SSEs (2009–2011 and 2018–2019). As a result, we found strong negative correlations for all four dilatations before and during SSE occurrence. For these dilatations, we estimated the amount of strain released at the time of occurrence of the SSE relative to that accumulated during the period prior to the SSE. The result indicates that not all the accumulated strain before the SSEs was released when the SSEs occurred. Moreover, it is highly likely that the strain released by SSE is not only due to the strain accumulation just below the SSE occurrence region, but also due to the strain accumulation on the shallower plate boundary, which is a seismogenic zone for a future megathrust earthquake.

## Introduction

**Figure 1:**
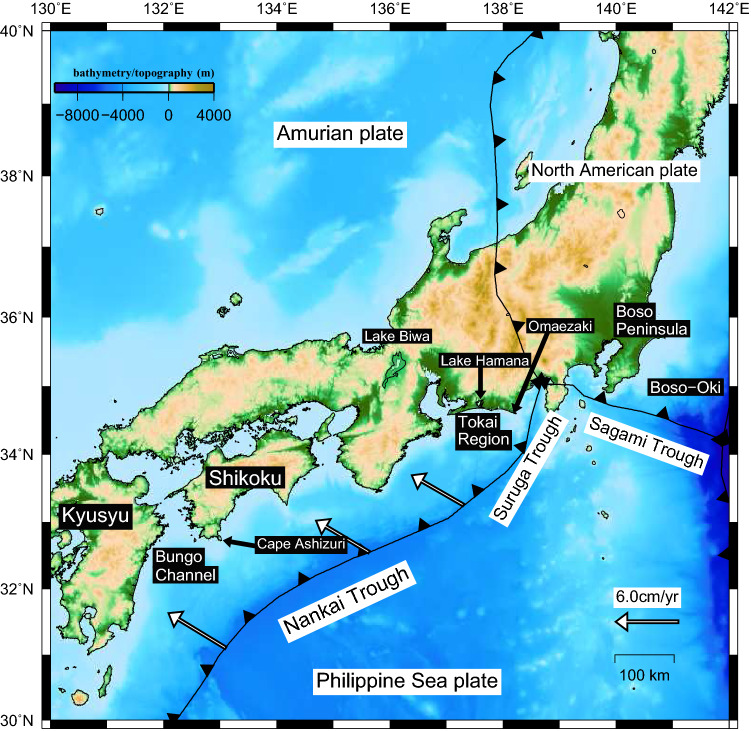
Tectonic map of the study area. The black barbed lines represent the plate boundary, and the triangles indicate the direction of motion of the subducting plate with respect to the plate being subducted (Bird^28^). The white arrows represent the velocity vectors of the Philippine Sea plate with respect to the Amurian plate (DeMets et al.^22^). The map was created by using the Generic Mapping Tools (GMT)^27^ (version: GMT4.5.7, URL link: https://www.generic-mapping-tools.org/download/).

Interplate earthquakes are considered to occur when the accumulated strain at the plate interface between a continental plate and a subducting oceanic plate exceeds a certain threshold, resulting in the release of the accumulated strain. Crustal deformation caused by the interaction between oceanic and continental plates is active in the Japanese Islands. Sagiya et al.^1^ investigated the strain rates (principal strain, dilatation, and maximum shear strain) of the Japanese Islands during 1997–1999 after removing coseismic steps and found that many regions are subject to contraction. In southwestern Japan, the oceanic Philippine Sea (PHS) plate is subducting in a northwesterly direction beneath the continental Amurian plate along the Suruga Trough to the Nankai Trough (Fig. 1). On the plate boundary, in addition to ordinary interplate earthquakes, interplate aseismic slow slip events, known as long-term slow slip events (hereafter referred to as L-SSEs), take place in the Tokai region and the Bungo Channel at durations of several months to several years.

L-SSEs with durations of several years have occurred in the Tokai region from 2000 to 2005 and from 2013 to 2016 and in the Bungo Channel from 1996 to 1997, 2003 to 2004, 2009 to 2011, and 2018 to 2019. Ozawa et al.^2^ and Miyazaki et al.^3^ reported southeastward slip as the L-SSE, which is distinct from the northwestward steady crustal movement, in the Tokai region from 2000 to 2005. Kobayashi and Hashimoto^4^ found strain changes in the area east of Lake Biwa from the latter half of 2000, which is considered to be due to the L-SSE in the Tokai region. Ozawa et al.^5,6^, Yoshioka et al.^7^, Nakata et al.^8^, Seshimo and Yoshioka^9^, and Agata et al.^10^ estimated the slip distribution of the L-SSEs in the Bungo Channel that occurred from 2009 to 2011 and from 2018 to 2019.

Short-term SSEs (hereafter referred to as S-SSEs), with a duration of several tens of days, have also occurred off the Boso Peninsula, where the PHS plate is subducting beneath the North American plate along the Sagami Trough with recurrence intervals of several years. According to Ozawa et al.^11^, Hirose et al.^12^, and Fukuda^13^, displacements caused by the Boso-Oki S‒SSEs were observed in the Boso Peninsula for approximately 10 days from approximately 10 August 2007. The Boso-Oki S‒SSE was included in this study because the displacement changes identified in GNSS time series data are more distinct than those of S-SSEs in other regions, and its recurrence interval is well known (e.g., Fukuda^13^).

Although many studies on L-SSEs and S-SSEs in the Japanese Islands have been conducted, a comprehensive relationship between the accumulation and release of strain before and during the occurrence of SSEs, respectively, has yet to be conducted. Strain accumulation and release are fundamental phenomena in seismology, make it very important to investigate them. One of the major characteristics of SSEs is that the interval between the end of the previous SSE and the onset of the next SSE is well known. This makes it possible to directly compare the amount of strain accumulated before the SSE with the amount of strain released during the SSE. On the other hand, although the earthquake cycles of large earthquakes are generally considered to range from several decades to several thousand years, GNSS data are currently available for only at most 25 years. Therefore, it is difficult to know in detail the accumulation of strain during the period between earthquakes and to directly compare the amount of strain accumulated before and that released by a large earthquake. Therefore, in this study, for these SSEs, we calculated the strain fields before and during the occurrence of SSEs and investigated the relationship between the accumulation and release of strain caused by one cycle of the SSEs based on their spatial distribution and the amount of strain accumulation and release using GNSS time series data.

## Results

The Tokai L-SSE (1 July 2000–30 June 2005), the Boso-Oki S‒SSE (9 August 2007–23 August 2007), and the two Bungo Channel L-SSEs (24 November 2009–6 February 2011 and 20 April 2018–26 May 2019) were the four SSEs analysed in this study. The former and the latter Bungo Channel L-SSE are hereafter referred to as the Bungo Channel L-SSE (1) and the Bungo Channel L-SSE (2), respectively. We selected the Tokai L-SSE and the Boso-Oki S‒SSE that occurred before the Tohoku earthquake (Mw 9.0) (11 March 2011) to avoid the influence of its postseismic effects. The Bungo Channel L-SSEs (1) and (2) are selected because many observation stations are available. For the Tokai L-SSE and the Bungo Channel L-SSE (1), (2), we used the results of previous studies (Suito and Ozawa^14^; Yoshioka et al.^7^; Seshimo and Yoshioka^9^) as a reference, dividing one year into every 0.1 year to estimate each L-SSE. We estimated the period from the onset to the end of the L-SSE. Since the occurrence time of the Boso-Oki S‒SSE is very short compared to other L-SSEs, we determined the onset and end dates of the S-SSEs, by checking the analysis results of Fukuda^13^ and the actual GNSS time series data. The number of observation stations used for each SSE and the respective analysis periods before and during the occurrence of SSE are tabulated in Table S1. The spatial distributions of GNSS stations used in the analysis of the four SSEs are shown in Fig. S1a–d.

In this study, the displacement fields for the four SSEs were obtained by GNSS time series analysis, and various strain fields (dilatation, maximum shear strain, and principal strain) were then obtained by the method of Shen et al.^15^ as described in the “Methods” section. For dilatation and maximum shear strain, correlation coefficients between the amount of strain accumulated before the SSE and the amount of strain released during the occurrence of SSE were calculated. Calculation points were the only metric used in spots where the amount of displacement during the SSE exceeded a threshold (Table S2). The threshold values for each SSE were obtained using the trial-and-error method, such that the negative correlation of dilatation, which specifically represents the accumulation and release of strain, is large. The distribution of calculation points used to calculate the correlation coefficient for each SSE is shown in Fig. S2a–d. For both dilatation and maximum shear strain, the stronger the negative correlation is, the greater the amount of strain release during the occurrence of SSE at locations where a large amount of strain accumulation before the SSE took place. This suggests that there is a strong relationship between strain accumulation and strain release.

In the following subsections, we show the results of the Bungo Channel L-SSE (1), (2). As for the results of the Tokai L-SSE and the Boso-Oki S‒SSE, please refer to Text S1 and S2, respectively in Supplementary Information.

### Bungo channel L-SSE (1)

**Figure 2:**
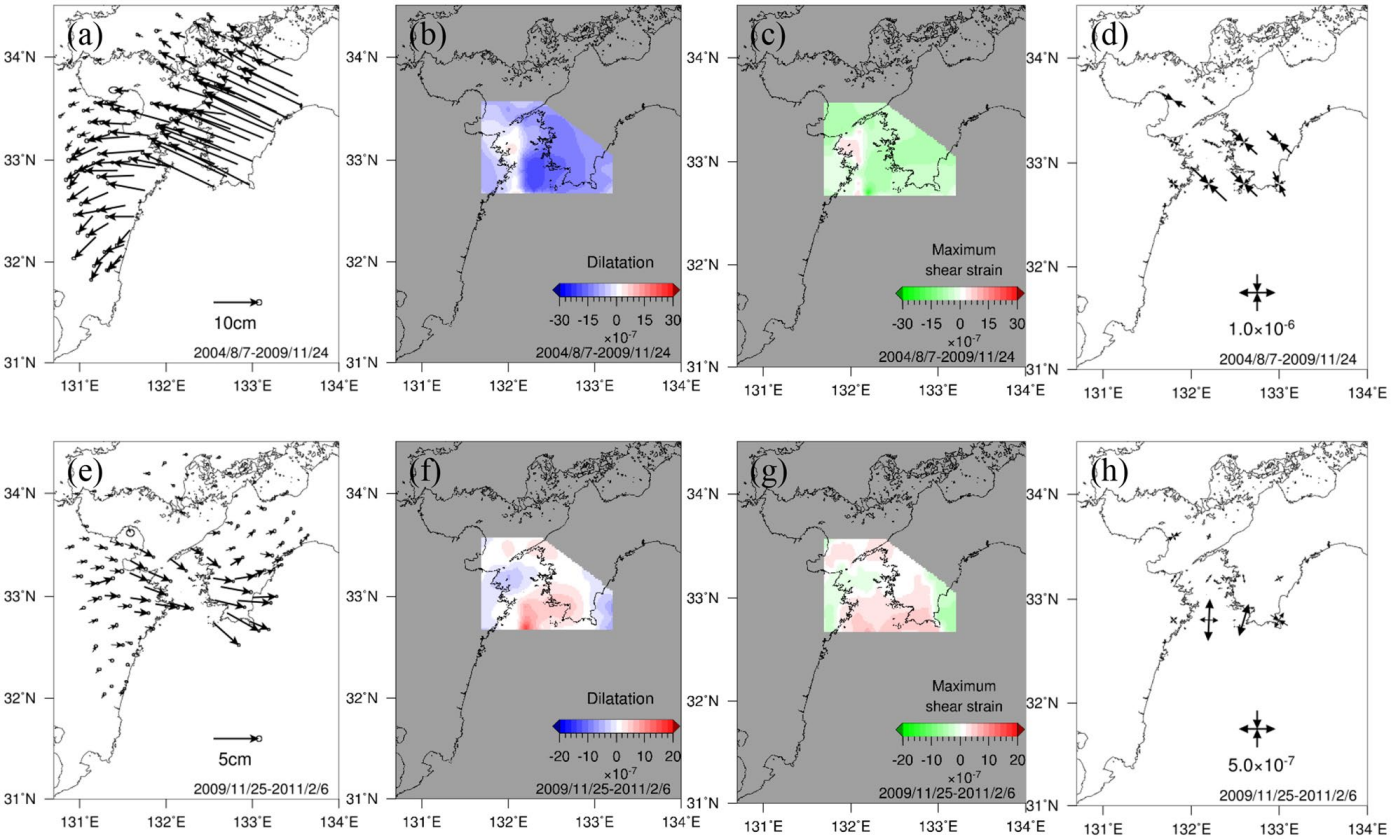
Spatial distributions of displacement, dilatation, maximum shear strain, and principal strain before (7 August 2004–24 November 2009) and during (25 November 2009–6 February 2011) the Bungo Channel L-SSE (1). (**a**)–(**d**) represent before the L-SSE, and (**e**)–(**h**) denote during the L-SSE. (**a**), (**e**) Spatial distribution of displacement. Black arrows show horizontal displacements during the analysis period. The ellipse at the tip of the black arrow represents the 1σ error ellipse. (**b**), (**f**) Spatial distribution of dilatation. The blue and red areas represent contraction and expansion, respectively. (**c**), (**g**) Spatial distribution of maximum shear strain. The green and red areas show compressive and tensile fields, respectively. (**d**), (**h**) Spatial distribution of principal strain. The black outwards and inwards arrows show tension and compression, respectively. The map was created by using the Generic Mapping Tools (GMT)^27^ (version: GMT3.4.6, URL link: https://www.generic-mapping-tools.org/download/).

The displacement, dilatation, maximum shear strain, and principal strain during the Bungo Channel L-SSE (1), which occurred from 25 November 2009 to 6 February 2011, are shown in Fig. 2a–d for the period before the L-SSE and are shown in Fig. 2e–h during the occurrence of the L-SSE. GNSS stations mainly in the Shikoku region show a northwestward displacement field before the L-SSE, while those in the Kyushu region show a gradual spatial change to the southwest. This may be partly due to the spatial change in plate interactions along the Nankai Trough (Sagiya et al.^1^).

The dilatation showed a characteristic contraction and expansion before and during the L-SSE, respectively, approximately 32.8° N on the south side of the Bungo Channel, and the maximum change in dilatation during the L-SSE occurrence was $8.2 \times 10^{ - 7}$.

For the maximum shear strain, negative and positive values are defined in this study. Positive values represent lateral deformation when the tensile component is dominant, while negative values denote lateral deformation when the compressive component is dominant. The region with large maximum shear strain was identified near the same region for dilatation during the L-SSE, and the values shifted from negative before the L-SSE to positive during the L-SSE. However, the location of the large strain release during the L-SSE moved slightly east to the west of Cape Ashizuri from the location of the large strain accumulation before the L-SSE. At the time of the L-SSE, the positive values were larger near the west side of Cape Ashizuri than the location where the expansion was identified in the dilatation.

With regard to principal strain, before the L-SSE, compression in the northwest‒southeast direction was dominant from western Shikoku to the Bungo Channel, but during the L-SSE, tension in the north‒northeast-south‒southwest direction was identified at approximately 32.8° N, where large changes in the dilatation and maximum shear strain can be seen. At other locations, no characteristic principal strain was observed. Comparing the amount of strain accumulated before the L-SSE and the amount of strain released during the L-SSE, the latter was generally smaller.

**Figure 3:**
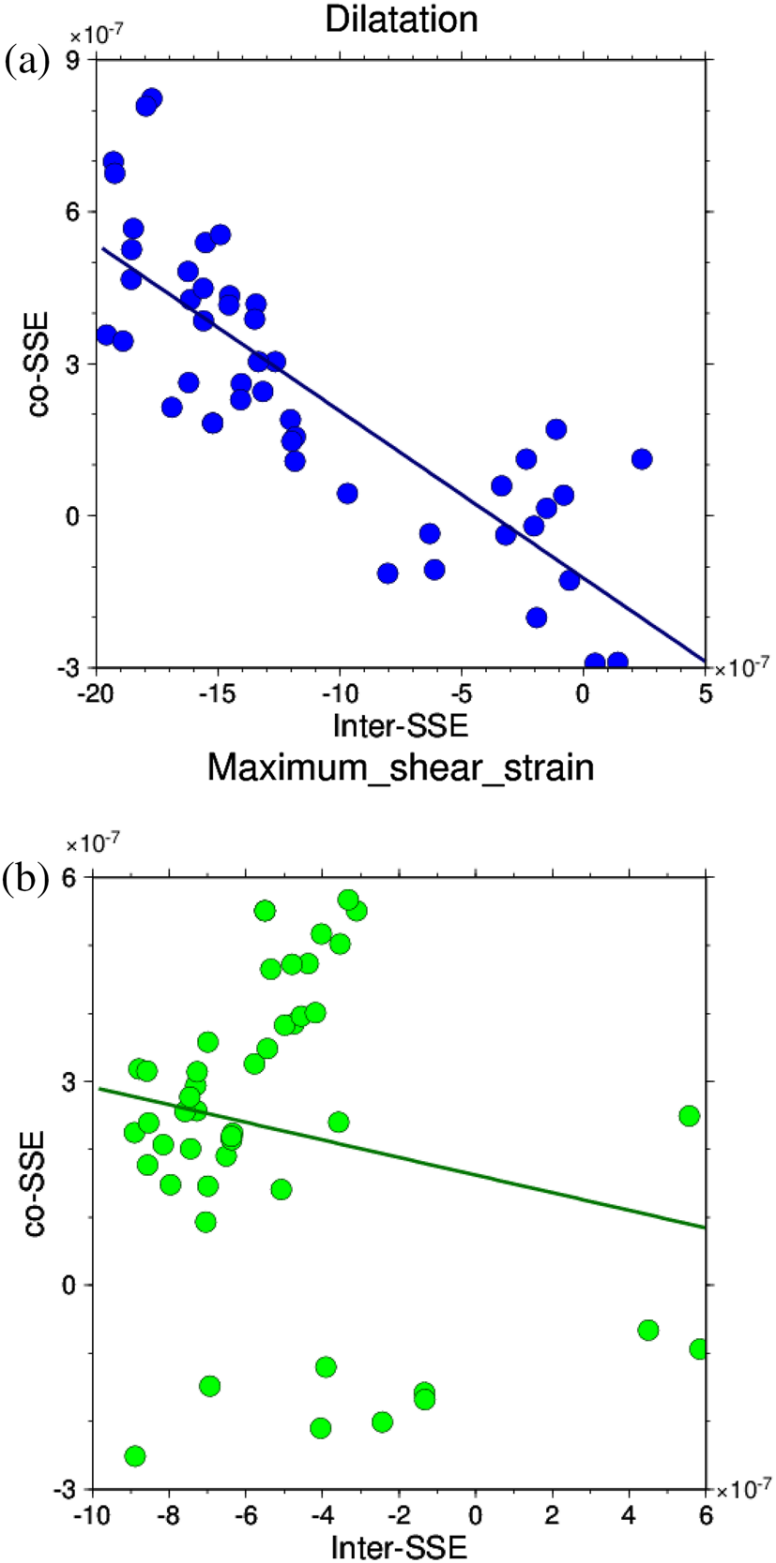
Correlation diagram between the amount of accumulated strain before the Bungo Channel L-SSE (1) (7 August 2004–24 November 2009) and the amount of change in strain during the L-SSE occurrence (25 November 2009–6 February 2011). The values of dilatation and the maximum shear strain at the calculation points shown in Fig. S2c are used. The straight line represents the linear regression line. (**a**) Dilatation. (**b**) Maximum shear strain.

Next, correlation coefficients were obtained for dilatation and maximum shear strain before and during the L-SSE (Fig. 3a and b). Fig. S2c shows the 48 calculation points used with a displacement of 2.5 cm or greater during the L-SSE. The correlation coefficient for dilatation was − 0.82, which is a strong negative correlation, while that for maximum shear strain was − 0.20, showing that the correlation was not as negative as that for dilatation. In the correlation diagram for the maximum shear strain (Fig. 3b), a positive correlation can be seen in the upper left area. This is because the release of maximum shear strain during the L-SSE occurred slightly east of where the maximum shear strain had accumulated before the L-SSE, as described above. As a result, the maximum shear strain did not show a stronger negative correlation than the dilatation. In conclusion, the Bungo Channel L-SSE (1) indicates that as for dilatation, the release of strain during the L-SSE is greater at locations where a greater accumulation of strain is identified before L-SSE.

### Bungo channel L-SSE (2)

**Figure 4:**
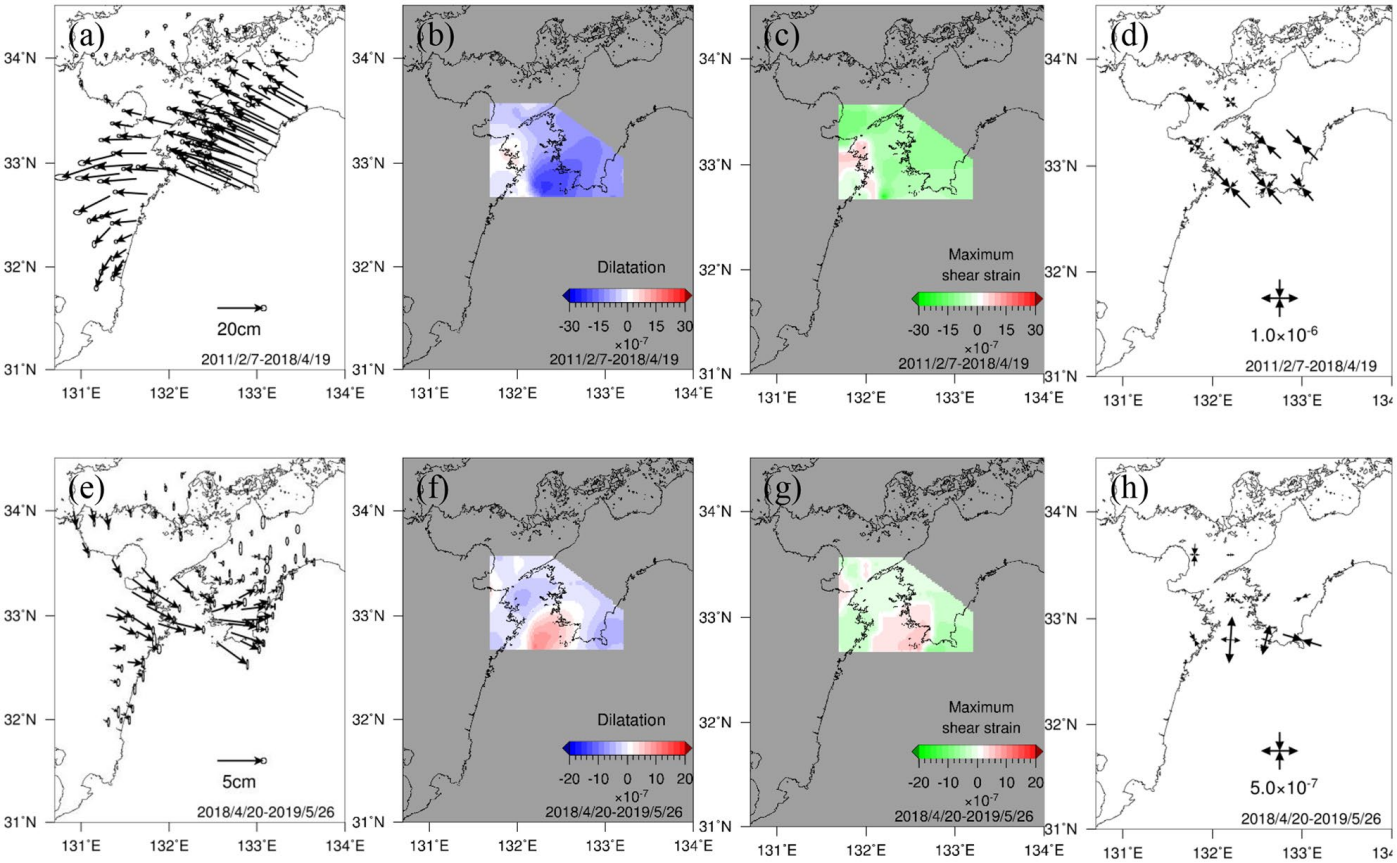
Spatial distributions of displacement, dilatation, maximum shear strain, and principal strain before (7 February 2011–19 April 2018) and during (20 April 2018–26 May 2019) the Bungo Channel L-SSE (2). The expressions of the figures are the same as those in Fig. 2a–h. The map was created by using the Generic Mapping Tools (GMT)^27^ (version: GMT3.4.6, URL link: https://www.generic-mapping-tools.org/download/).

The displacement, dilatation, maximum shear strain, and principal strain for the Bungo Channel L-SSE (2) that occurred from 20 April 2018 to 26 May 2019 are shown in Fig. 4a–d for the period before the L-SSE and in Fig. 4e–h during the L-SSE. The GNSS stations in western Shikoku show the northwesterly displacement field, while those in the southeastern Kyushu region show a gradual spatial change to the southwesterly direction, as in the previous L-SSE (1). The displacements for the accumulation period of the L-SSE (2) (Fig. 4a) were larger than those of the L-SSE (1) (Fig. 2a) because of the longer analysis period before L-SSE (2). During L-SSE (2), both the direction and magnitude of displacement were similar to those of L-SSE (1).

The results of the dilatation of L-SSE (2) were also similar to those of L-SSE (1), with characteristic contraction and expansion before and during L-SSE at approximately 32.8° N, respectively, and a maximum amount of dilatation of approximately $8.8 \times 10^{ - 7}$ during the occurrence of L-SSE.

The region with a large maximum shear strain was identified at the time of the L-SSE (2), and the values turned from negative before the L-SSE to positive during the L-SSE. As in the case of L-SSE (1), the location of the large strain release during L-SSE (2) shifted slightly east of the location of the large strain accumulation before L-SSE. At the time of the L-SSE (2), positive values on the west side of the Bungo Channel were larger than positive values around the Bungo Channel. However, the location of the spatial boundary between the positive area and negative area on the west side of Cape Ashizuri was slightly shifted to the west during L-SSE (2) than during L-SSE (1).

The compressive principal strain was dominant in the northwest‒southeast direction before L-SSE (2), whereas during L-SSE, extension in the north‒northeast-south‒southwest direction was dominant. Comparing the amount of accumulated strain before the L-SSE with the amount of strain released during the L-SSE, the latter was smaller than the former.

**Figure 5:**
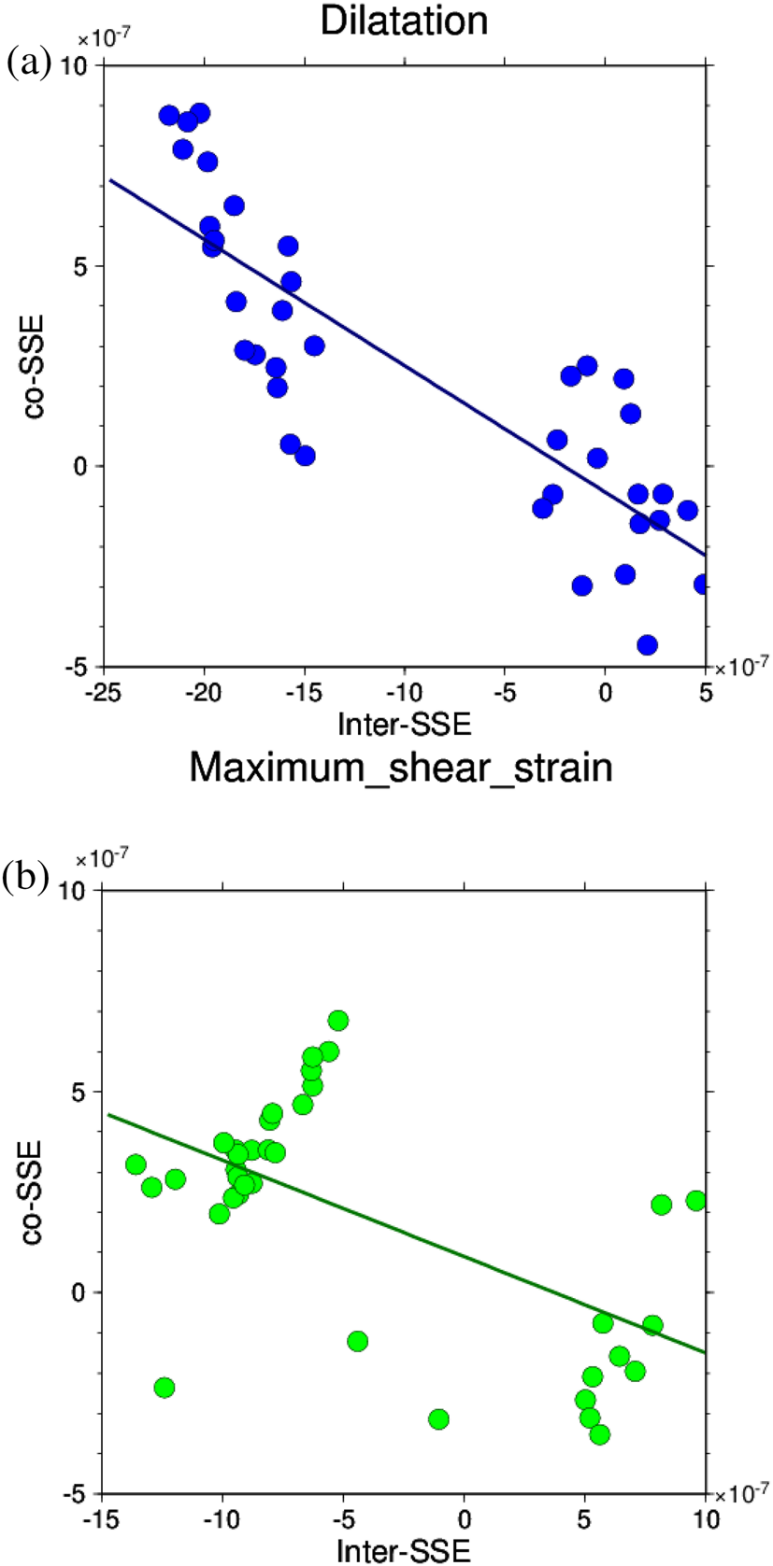
Correlation diagram between the amount of strain accumulated before the Bungo Channel L-SSE (2) (7 February 2011–19 April 2018) and the amount of change in strain during the L-SSE occurrence (20 April 2018–26 May 2019). The values of the dilatation and the maximum shear strain at the calculation points shown in Fig. S2d are used. The expressions of the figures are the same as those in Fig. 3a and b.

Figure 5a and b show the correlations for dilatation and maximum shear strain, respectively, for L-SSE (2). Fig. S2d shows 37 used calculation points with a displacement of 2.8 cm or greater during the L-SSE. As with the L-SSE (1), the dilatation showed a strong negative correlation with a correlation coefficient of − 0.84. That of the maximum shear strain is − 0.59, indicating a negative correlation similar to that of the Boso-Oki S‒SSE (Text S2). However, Fig. 5b does not show a clear downwards trend to the right, as in the case of dilatation. As with L-SSE (1), this is because the location of the large maximum shear strain moved slightly to the east at the time of the L-SSE compared to that before the L-SSE, and the relationship between the accumulation and release of maximum shear strain is considered to be small. These results indicate that for the Bungo Channel L-SSE (2), a large amount of expansion was released when the L-SSE occurred, where the accumulation of contraction was also large before the occurrence of L-SSE in the dilatation.

## Discussion

### Estimation of the number of years of strain accumulation before each SSE

From the results described in the previous section, strong negative correlations in the dilatation were identified for Tokai L-SSE (Text S1), Boso-Oki S‒SSE (Text S2), and Bungo Channel L-SSEs (1) and (2) (Table S3). In this section, we estimate the number of years of strain accumulation for these four SSE dilatations with large negative correlations.

Here, the number of years of strain accumulation before a SSE is defined as the number of years required to accumulate an amount of strain equivalent to the change in strain released by the SSE. The following Eqs. (1) and (2) were used to estimate the number of years of strain accumulation $T$. For this calculation, the same calculation points were used as those used for the calculation of the correlation coefficients.

1 $$\begin{array}{*{20}c} {RMS = \sqrt {\frac{{\mathop \sum \nolimits_{i = 1}^{n} \left( {\left| {e_{i}^{eq} } \right| - t\left| {e_{i} } \right|} \right)^{2} }}{n}} } \\ \end{array}$$

2 $$\begin{array}{*{20}c} {T = tT_{0} } \\ \end{array}$$

where $e_{i}^{eq}$ is the amount of released dilatation during the SSE at the $i$-th calculation point, $e_{i}$ is the amount of strain accumulated at the $i$-th calculation point in the period before the SSE, *n* is the number of calculation points, and the value of $t$ that minimizes the RMS value is obtained by the least-squares method. In Eq. (1), the smaller the value of RMS is, the better the absolute value of the released strain $e_{i}^{eq}$ and the product of the absolute value of the accumulated strain $e_{i}$ and $t$ match. In Eq. (2), $T_{0}$ is the period before the occurrence of each SSE set in this study, and the accumulated years $T$ were obtained by taking the product of the value of $t$ when the value of RMS was at its minimum in Eq. (1). The optimal value for the number of years of strain accumulation was searched by changing the values of $t$ in the range from 0.01 to 10.00 at intervals of 0.01.

Table 1 shows the estimated number of years of strain accumulation for each SSE. For the Boso-Oki S‒SSE and the Bungo Channel L-SSEs (1) and (2), for which the end of the previous SSE is known, the estimated accumulation years are 1.7 years for the Boso-Oki S‒SSE, 1.4 years for the Bungo Channel L-SSE (1), and 2.1 years for the Bungo Channel L-SSE (2). Considering that the analysis periods before these SSEs were 4.7, 5.3, and 7.2 years, respectively, the accumulation years for the Boso-Oki S‒SSE, Bungo Channel L-SSE (1), and Bungo Channel L-SSE (2) were all approximately 30% of the total years. This indicates that approximately 30% of the strains accumulated prior to the SSEs being released during the SSEs.

**Table 1:** Years of strain accumulation at each SSE.

SSE (Duration)	Estimated years of strain accumulation (Analysis period prior to the SSE) (years)
Tokai L-SSE (1 July 2000–30 June 2005)	4.69 (3.50)
Boso-Oki S-SSE (9 August 2007–23 August 2007)	1.65 (4.72)
Bungo Channel L-SSE (1) (25 November 2009–6 February 2011)	1.38 (5.30)
Bungo Channel L-SSE (2) (20 April 2018–26 May 2019) (Before correction of coseismic steps)	2.09 (7.20)
Bungo Channel L-SSE (2) (20 April 2018–26 May 2019) (After correction of coseismic steps)	1.33 (5.12)

The values in parentheses in the right column represent the analysis period prior to the SSEs, whereas the values without parentheses in the right column denote the accumulation years calculated by the method described in the section of “Discussion”.

For the Tokai L-SSE, the estimated accumulation period was 4.7 years, which is longer than 3.5 years of the analysis period before the L-SSE. This longer period is because the installation of GNSS stations in the Tokai region started on 21 March 1996, and we were unable to observe L-SSEs that ended before that date in the region. However, there were reports that crustal deformations equivalent to L-SSE were observed in the Tokai region before 2000, without using GNSS data. Fujii^16^ pointed out that, based on the measured side lengths in the Tokai region, an extension different from the constant east–west compression was observed in the area from Lake Hamana to Omaezaki in approximately 1974. Ohtake and Asada^17^ reported unusual crustal deformation in the Omaezaki area in 1976 based on observations of tidal level changes, radon concentrations in water, and strain gauges operated by the Japan Meteorological Agency. Kimata et al.^18^ stated that L-SSEs occurred from 1978 to 1983 and 1987 to 1991 based on the analysis of laser ranging and levelling. Kobayashi and Yoshida^19^ noted that similar changes occurred in 1980–1982 and 1988–1990 based on their analysis of tidal records, suggesting that L-SSEs may have occurred. Yamamoto et al.^20^ reported that an L-SSE occurred between 1988 and 1990 based on an analysis of tilt change. Based on these previous studies, we can conjecture that crustal deformations similar to the 2000–2005 L-SSE occurred in the Tokai region in the late 1970s and late 1980s, with a recurrence interval of approximately 10 years, although there was some variance during the period. If the end year of the previous L-SSE that took place before the L-SSE analysed in this study was assumed to be 1990, the period before the L-SSE was approximately 10 years. For the Tokai L-SSE that initiated in 2000, considering that the accumulation period of dilatation was 4.7 years, approximately 50% of the strain accumulated before its occurrence was released when the L-SSE occurred.

### The relationship between slip caused by SSEs and interplate coupling

In this subsection, we discuss the relationship between interplate coupling and release due to slip at the plate boundary based on the above strong spatial relationship in the dilatation between the accumulation before the SSE and release during the SSE at the Earth’s surface. Here, we consider the Bungo Channel L-SSE as an example, where approximately 30% of the accumulated strain was released during the L-SSE. According to Seshimo and Yoshioka^9^, in many areas of the plate boundary beneath the Bungo Channel, long-term interplate coupling is more dominant than release caused by slip associated with L-SSEs. In terms of the displacement field at the Earth’s surface, from Figs. 2a and e and 4a and e, the amount of displacement in the northwest direction before the L-SSE is much larger than the amount of displacement during the L-SSE.

**Figure 6:**
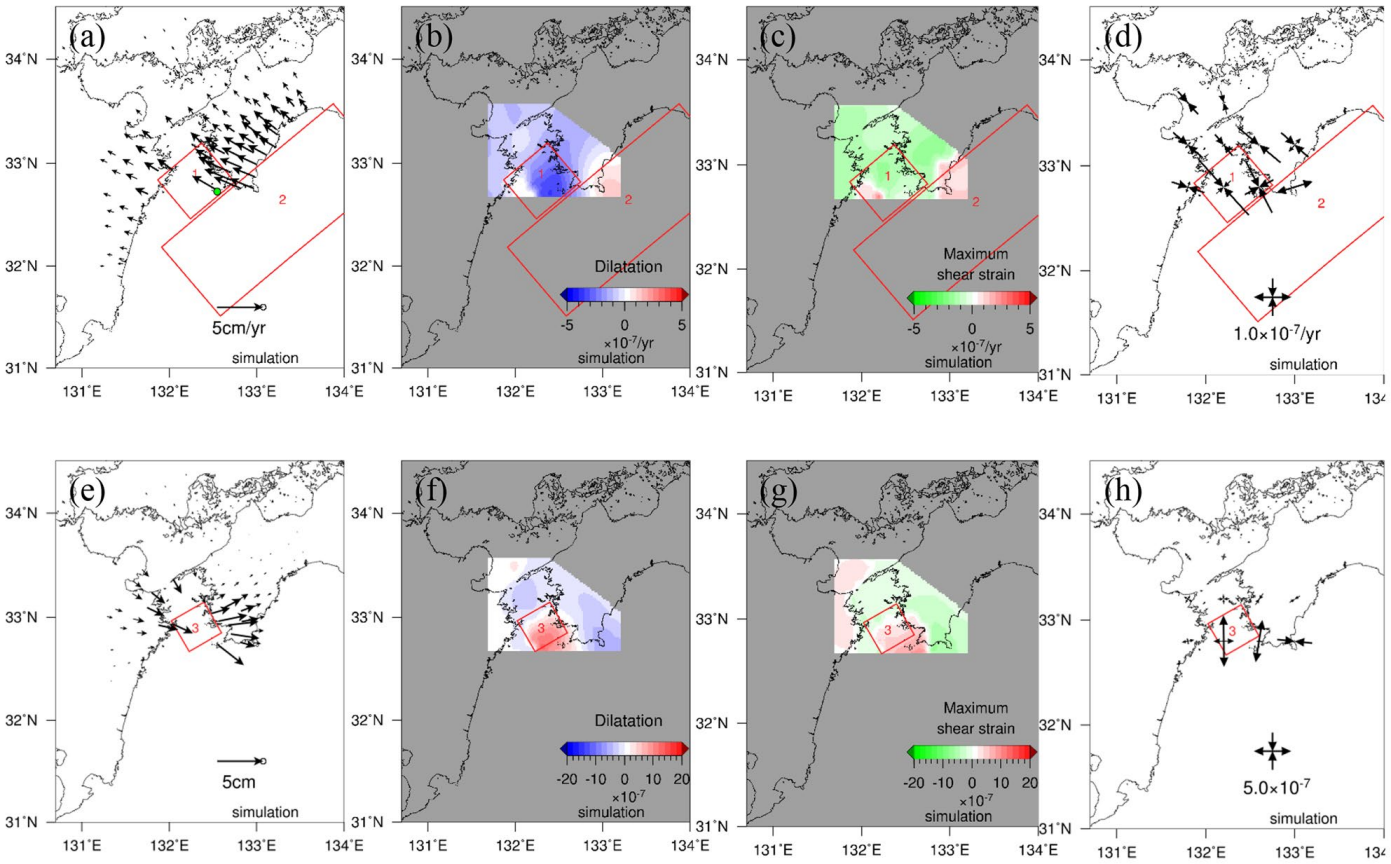
Displacement and various strain fields at the Earth’s surface calculated using the rectangular fault model beneath the Bungo Channel and its shallower plate interface. Red rectangles 1 to 3 represent the horizontal projections of the assumed fault planes (see Table 2). The green dot in (**a**) is the Sukumo station (021059), which is the closest to the Bungo Channel. The map was created by using the Generic Mapping Tools (GMT)^27^ (version: GMT3.4.6, URL link: https://www.generic-mapping-tools.org/download/).

To explain the result of 30% strain release relative to the amount of strain accumulation, we discussed the relationship between interplate coupling (or slip deficit) and forwards slip related to strain release at the plate boundary. In this discussion, we attempted to find a model that can explain the displacement fields observed in the Bungo Channel during the accumulation and release periods by forward modelling, using a static fault model in a semi-infinite homogeneous perfect elastic body (DC3D) (Okada^21^). Here, we used the accumulation and release periods of the 2018–2019 Bungo Channel L-SSE, with a displacement of 3.3 cm/yr for the accumulation period at the GNSS station closest to the Bungo Channel (Fig. 6a). For interplate coupling, the slip deficit in the direction of plate convergence by DeMets et al.^22^ was given on a rectangular fault plane at the plate boundary placed directly beneath the Bungo Channel. For strain release, forward slip in the opposite direction to that of DeMets et al.^22^ was given on a rectangular fault on the plate interface directly beneath the Bungo Channel.

As a result, we found that the observed displacement field was not well reproduced by providing a slip deficit only immediately beneath the Bungo Channel. However, by also providing the interplate coupling of the shallower side, namely, the seismogenic zone, in addition to the coupling beneath the Bungo Channel, we were able to reproduce the observed displacement field. This is consistent with the results of Yokota et al.^23^ and Noda et al.^24^, who estimated relatively strong interplate coupling on the shallow side of the plate boundary. Thus obtained displacement and various strain fields are shown in Fig. 6a–d. The slip deficit rate is 6.2 cm/yr in both the Bungo Channel and the shallower seismogenic zone, which indicates a coupling ratio of 1.0 compared with the convergence rate by DeMets et al.^22^. This implies acoupling of 6.2 (cm/yr) × 7.2 (yr) ≈ 44.6 cm during the period of strain accumulation prior to the L-SSE occurrence. On the other hand, for the strain release process, a forward slip of 28.0 cm was given, which was very close to the observed displacement field associated with the L-SSE. The displacement field and various strain fields are shown in Fig. 6e–h. Each fault parameter used for these calculations is also tabulated in Table 2.

**Table 2:** Fault parameters for the three rectangular faults shown in Fig. 6.

Parameter	Fault 1	Fault 2	Fault 3
Length (km)	62.0	240.0	40
Width (km)	57.5	100.0	40
Dislocation (cm)	6.2	6.2	28.0
Depth to the upper edge of the fault plane (km)	20.0	10.0	20.0
Strike (°)	230.0	230.0	240.0
Dip (°)	17.5	12.5	17.5
Rake (°)	286.3	287.4	116.3

Therefore, by comparing 44.6 cm during the accumulation period and 28.0 cm during the release period, the ratio of release to accumulation is approximately 60%, which means that 60% of the interplate coupling at the plate boundary beneath the Bungo Channel and its shallow seismogenic zone slipped beneath the Bungo Channel. This value is large compared to the dilatation release rate of 30%. However, this value is likely due to the smaller limited forward slip area beneath the Bungo Channel compared to the large strongly coupled area at the plate interface both in the shallow seismogenic zone and the Bungo Channel, resulting in the larger value of the forward slip/slip deficit ratio. We also confirmed that the ratio of strain accumulation and release at this time was calculated to be approximately 30% of the total release, so this amount of slip appears to be reasonable.

In this case, the correlation coefficient in dilatation became -0.93, which is slightly larger than the value obtained in the strain analysis. However, this may be due to neglecting the northwestwards to southwards displacements in the Kyushu region before the L-SSE occurrence (Figs. 2a, 4a) in the observed value. This effect may have reduced the correlation coefficient to approximately − 0.8 for the observed value. In addition, the poor correlation of the maximum shear strain (Fig. 6c, g) and why the principal strain is not in the opposite direction before and during the L-SSE occurrence (Fig. 6d, h) can be explained by forward slip associated with the L-SSE only beneath the Bungo Channel at the time of strain release.

Therefore, we attempted to reproduce the strain field in the Bungo Channel using DC3D and found that the observed values were best explained when a coupling ratio of 1.0 was applied to both the shallow part of the plate boundary and beneath the Bungo Channel. This finding suggests that interplate coupling in the shallow part may also contribute significantly to the accumulation of strain in and around the Bungo Channel.

## Conclusions

In this study, the spatial distribution of the strain field (dilatation, maximum shear strain, and principal strain) during the periods before and during the Tokai L-SSE, the Boso-Oki S‒SSE, and the Bungo Channel L-SSE (1) and (2) were obtained by analysing GNSS time series data. The significant results obtained in this study are summarized as follows:

1. In the Tokai L-SSE, the Boso-Oki S‒SSE, and the Bungo Channel L-SSE (1) and (2), in the dilatation, larger accumulation and release were observed on the Earth's surface in the area where the SSE occurred before and at the time of occurrence, respectively. In all the SSEs, the spatial distribution of the strain accumulation region before the SSE and the strain release region at the time of the SSE were in good agreement.
2. For the Boso-Oki S‒SSE and the Bungo Channel L-SSE (1) and (2), approximately 30% of the dilatation was released compared to the amount of strain accumulated before the SSE. In the Tokai L-SSE, approximately 50% of the strain release was identified when the strain accumulation period before the L-SSE was assumed to be 10 years. For the Bungo Channel L-SSE (2), the period required for strain accumulation was increased due to the 2011 Tohoku-Oki earthquake and the 2016 Kumamoto earthquake (Text S3). This increase indicates that some of the strain that had been accumulating before the Bungo Channel L-SSE (2) was released by the occurrences of both earthquakes.
3. The meaning of the ratio of strain release to accumulation at the Earth's surface was considered in terms of ratio of the forward slip to slip deficit at the plate boundary by forward modelling. As a result, we found that approximately 60% of the interplate coupling at the plate boundary beneath the Bungo Channel and its shallow seismogenic zone was released by forward slip only beneath the Bungo Channel.

## Methods

### Data processing of time series data

In this study, we used the daily coordinate positioning values (F5 solutions) of GNSS Earth Observation Network System (GEONET) stations provided by the Geospatial Information Authority of Japan (GSI), and the following data processing was performed for the time series.

Displacement is defined as the displacement relative to a distant reference station that is not affected by an SSE to confirm consistency with the direction of plate convergence. For the case of the Bungo Channel L-SSE (1) and (2), we used six reference stations, following Yoshioka et al.^7^ and Seshimo and Yoshioka^9^. Except for the Tokai L-SSE, the strain accumulation period before the SSE was defined as the period from the end of the previous SSE to just before the onset of the target SSE, and the period during the SSE was defined as the period from the onset of the SSE to its end. For the Tokai L-SSE, the starting date of the period before the SSE was set as 1 January 1997 because the GNSS time series data did not include the period of the previous Tokai L-SSE. This is because the observations by the GNSS stations operated by the GSI started on 21 March 1996. In this study, only the east–west and north–south components of horizontal displacement time series data were used.

The analytical procedure of GNSS time series data is described below, following the method of Abe and Yoshioka^25^. Note that the GNSS time series data are processed differently before and during the occurrence of an SSE: We investigated the accumulation of strain due to all crustal deformation, including steady crustal deformation and coseismic steps, whereas the release of strain was due to only the occurrence of the SSE. GNSS time series data include steps due to antenna exchanges, coseismic steps, annual and semiannual variations, variations due to SSEs, and common-mode error. In this study, to obtain the strain field due to crustal deformation, including an SSE, we first corrected the steps due to antenna exchanges from the original time series. The steps were corrected by taking the difference in the averages of the previous and next 10 days when a step of 0.5 cm or more was detected in the east–west or north–south component at a certain observation station. Next, curve fitting was performed on the corrected GNSS time series data using Eq. (3).

3 $$y\left( t \right) = a_{0} + \mathop \sum \limits_{i}^{n} a_{i} T_{i} \left( t \right) + b\sin \left( {\frac{2\pi t}{T}} \right) + c\cos \left( {\frac{2\pi t}{T}} \right) + d\sin \left( {\frac{4\pi t}{T}} \right) + e\cos \left( {\frac{4\pi t}{T}} \right) + \mathop \sum \limits_{k = 1}^{{N_{eq} }} f_{k} H\left( {t - t_{k}^{eq} } \right)$$

4 $$\begin{gathered} T_{1} \left( t \right) = t \hfill \\ T_{2} \left( t \right) = 2t^{2} - 1 \hfill \\ \begin{array}{*{20}c} \begin{gathered} T_{3} \left( t \right) = 4t^{3} - 3t \hfill \\ \vdots \hfill \\ \end{gathered} \\ \end{array} \hfill \\ \end{gathered}$$

where $t$ is the elapsed time from the analysis start date, $y\left( t \right)$ is the approximate curve, and $T$ = 1 year. The first and second terms on the right-hand side of Eq. (3) represent crustal deformation, and the second term in Eq. (3), $T_{i} \left( t \right)$, is a Chebyshev polynomial, as shown in Eq. (4). $n$ is varied from 1 to 20, and the optimal value of $n$ was determined using the AIC minimization principle (Akaike^26^). The third to sixth terms on the right-hand side of Eq. (3) denote the annual and semiannual variations. The seventh term represents coseismic steps caused by large earthquakes. $t_{k}^{eq}$ is the date of the $k$ th earthquake, and $H\left( {t - t_{k}^{eq} } \right)$ is a Heaviside step function. The unknown parameters $a_{0}$, $a_{i}$, $b$, $c$, $d$, $e,$ and $f_{k}$ were determined by the least-squares method. The residuals of the east–west and north–south components were obtained between the approximate curves and the step-corrected GNSS time series data. The residuals were obtained at each observation station and averaged over all observation stations used in the analysis except the reference stations to obtain the common-mode error. The common-mode error was removed from the GNSS time series data corrected for the steps due to antenna exchanges before the SSE, and then the curve fitting was performed again using Eq. (3). Likewise, the time series data at the time of an SSE were corrected for the steps due to antenna exchanges, and the curve fitting was performed again using Eq. (3). In this case, the unknown parameters for the annual and semiannual variations were fixed, using the obtained values calculated in the analysis before the SSE because the analysis period was much shorter than that before the SSE. The same analysis was performed for the reference stations, which were used only for the purpose of plotting the displacement field before and during the SSE, and the average GNSS time series data of the reference stations were subtracted from the GNSS time series data of each observation station. By accurately estimating the difference between the GNSS time series data at the start and end dates of each analysis, we used the above curve fitting.

### Analysis of the strain field

We analysed the strain field according to the coordinate system of the International Terrestrial Reference Frame 2014 (ITRF2014), and the time series data at the reference stations were not used. In this study, the method of Shen et al.^15^ was used to calculate the strain field. To obtain the strain field, the calculation points were placed on a grid interval of 0.1° over the area including the GNSS observation stations used, and the strain value was obtained at each of these points. According to Shen et al.^15^, we obtained the strain field, as described below.

5 $$\begin{array}{*{20}c} {\left( {\begin{array}{*{20}c} {U_{x}^{i} } \\ {U_{y}^{i} } \\ \end{array} } \right) = \left( {\begin{array}{*{20}c} 1 & {\quad 0} & {\quad \Delta x_{i} } & {\quad \Delta y_{i} } & {\quad 0} & {\quad \Delta y_{i} } \\ 0 & {\quad 1} & {\quad 0} & {\quad \Delta x_{i} } & {\quad \Delta y_{i} } & {\quad - \Delta x_{i} } \\ \end{array} } \right)\left( {\begin{array}{*{20}c} {u_{x} } \\ {u_{y} } \\ {e_{xx} } \\ {e_{xy} } \\ {e_{yy} } \\ \omega \\ \end{array} } \right) + \left( {\begin{array}{*{20}c} {\varepsilon_{x}^{i} } \\ {\varepsilon_{y}^{i} } \\ \end{array} } \right)} \\ \end{array}$$

6 $$\begin{array}{*{20}c} {\varepsilon_{x,y}^{i} = \sigma_{x,y}^{i2} \exp \left( {\frac{{\Delta x_{i}^{2} + \Delta y_{i}^{2} }}{{D^{2} }}} \right)} \\ \end{array}$$

Although Shen et al.^15^ used the equation to obtain the strain rate, in this study, the total strain accumulation and release for each analysis period were obtained. Equation (5) was used to obtain the value of strain at the calculation points. $x$ and $y$ are the east–west and north–south components, respectively, with east and north as positive directions.$i$ is the observation point,$U_{x}^{i}$ and $U_{y}^{i}$ are the displacements at the GNSS station, $\Delta x_{i}$ and $\Delta y_{i}$ are the distances between the GNSS station and the calculation point, $u_{x}$ and $u_{y}$ are the displacements at the calculation points, $\omega$ is the rotation of the rigid body, $e_{xx}$, $e_{yy}$, and $e_{xy}$ are the strains at the calculation points to be obtained, $e_{xx}$ and $e_{yy}$ are the normal strains, and $e_{xy}$ is the shear strain. The second term on the right-hand side, $\varepsilon_{x}^{i}$ and $\varepsilon_{y}^{i}$, represents the observation error and decay over distance and is obtained by Eq. (6). In Eq. (6), $\sigma_{x}^{i}$ and $\sigma_{y}^{i}$ represent the observation error, which is given by the obtained standard deviation when determining the displacement at the GNSS station, performing the curve fitting using Eq. (3). $D$ represents the Distance Decaying Constant (DDC), and Table S4 shows values of the distance decaying constants used for each SSE. In this study, for each calculation point, GNSS observation points within a radius of $2D$ centred on the calculation point were used.

We used the strains ($e_{xx}$, $e_{yy}$, and $e_{xy}$) to obtain the dilatation, maximum shear strain, and principal strain.

7 $$\begin{array}{*{20}c} {e_{d} = e_{xx} + e_{yy} } \\ \end{array}$$

8 $$\begin{array}{*{20}c} {e_{s} = \sqrt {\frac{{\left( {e_{xx} - e_{yy} } \right)^{2} }}{4} + e_{xy}^{2} } } \\ \end{array}$$

9 $$\begin{array}{*{20}c} {e_{1,2} = \left( {e_{d} \pm e_{s} } \right)/2} \\ \end{array}$$

Equations (7) and (8) are used to determine the dilatation and maximum shear strain, $e_{d}$ and $e_{s}$, respectively. The dilatation is a strain that represents an increase or decrease in an area, with a positive value indicating expansion and a negative value indicating contraction. The maximum shear strain represents the degree of lateral deformation and varies in magnitude depending on the axis taken, but the maximum value is obtained. From the values of dilatation and maximum shear strain, the principal strains $e_{1}$ and $e_{2}$ are obtained using Eq. (9). The principal strain is expressed in terms of the strain in the two principal axes, and the direction of the principal axes, $\theta$, is determined by the following Eq. (10).

10 $$\begin{array}{*{20}c} {\theta = \frac{1}{2}\tan^{ - 1} \left( {\frac{{2e_{xy} }}{{e_{xx} - e_{yy} }}} \right)} \\ \end{array}$$

where $\theta$ is the azimuth of $e_{2}$ with north as the reference azimuth $0^\circ$ and positive values in the clockwise direction. Although the maximum shear strain originally takes only positive values, in this study, the maximum shear strain is redefined to take negative values to see the relationship between strain accumulation and release. From the two principal strains $e_{1}$ and $e_{2}$, when $\left| {e_{1} } \right| \ge \left| {e_{2} } \right|$,

11 $$\begin{array}{*{20}c} {e_{s} = e_{1} - e_{2} } \\ \end{array}$$

and when $$\left| {e_{1} } \right| < \left| {e_{2} } \right|$$,

12 $$\begin{array}{*{20}c} {e_{s} = e_{2} - e_{1} } \\ \end{array}$$

Equation (11) represents the maximum shear strain at the calculation points where the tensile component of the principal strain dominates, where the maximum shear strain is positive. Equation (12) represents the maximum shear strain at the calculation points where the compressive component of the principal strain is dominant and negative. The above three types of strains were calculated for the period before and during the occurrence of each SSE.

## Supplementary Information

Supplementary Information.

## Data Availability

We used the daily coordinate data of GNSS stations provided by the Geospatial Information Authority of Japan (GSI) (https://www.gsi.go.jp/ENGLISH/geonet_english.html) for displacement, the electronic reference point maintenance work list (https://terras.gsi.go.jp/denshi_hosyu.php), and JMA's seismic intensity database (https://www.data.jma.go.jp/svd/eqdb/data/shindo/index.html). The figures were created using the Generic Mapping Tools (version: GMT 3.4.6, URL link: https://www.generic-mapping-tools.org/download/) by Wessel and Smith^27^.

## References

[1] Sagiya T, Miyazaki S, Tada T (2000). Continuous GPS array and present-day crustal deformation of Japan. Pure Appl. Geophys..

[2] Ozawa S (2002). Detection and monitoring of ongoing aseismic slip in the Tokai region, central Japan. Science.

[3] Miyazaki S, Segall P, McGuire JJ, Kato T, Hatanaka Y (2006). Spatial and temporal evolution of stress and slip rate during the 2000 Tokai slow earthquake. J. Geophys. Res..

[4] Kobayashi T, Hashimoto M (2007). Change of strain rate and seismicity in the Chubu district, central Japan, associated with a Tokai slow event. Earth Planets Space.

[5] Ozawa S, Yarai H, Imakiire T, Tobita M (2013). Spatial and temporal evolution of the long-term slow slip in the Bungo Channel, Japan. Earth Planets Space.

[6] Ozawa S, Kawabata R, Kokado K, Yarai H (2020). Long-term slow slip events along the Nankai trough delayed by the 2016 Kumamoto earthquake Japan. Earth Planets Space.

[7] Yoshioka S, Matsuoka Y, Ide S (2015). Spatiotemporal slip distributions of three long-term slow slip events beneath the Bungo Channel, southwest Japan, inferred from inversion analyses of GPS data. Geophys. J. Int..

[8] Nakata R (2017). Discontinuous boundaries of slow slip events beneath the Bungo Channel, southwest Japan. Sci. Rep..

[9] Seshimo Y, Yoshioka S (2022). Spatiotemporal slip distributions associated with the 2018–2019 Bungo Channel long-term slow slip event inverted from GNSS data. Sci. Rep..

[10] Agata, R. et al, Bayesian multi-model estimation for fault slip distribution in slow slip events in southwest Japan focused on prior constraints and uncertain underground structure. *J. Geophys. Res. Solid Earth*, **127** (2022).

[11] Ozawa S, Suito H, Tobita M (2007). Occurrence of quasi-periodic slow-slip off the east coast of Boso Peninsula, central Japan. Earth Planets Space.

[12] Hirose H, Kimura H, Enescu B, Aoi S (2012). Recurrent slow slip event likely hastened by the 2011 Tohoku earthquake. Proc. Natl. Acad. Sci. U.S.A..

[13] Fukuda J (2017). Variability of the space-time evolution of slow slip events off the Boso Peninsula, central Japan, from 1996 to 2014. J. Geophys. Res.: Solid Earth.

[14] Suito H, Ozawa S (2009). Transient crustal deformation in the Tokai district—The Tokai slow slip event and postseismic deformation caused by the 2004 off southwest Kii peninsula earthquake. Zisin.

[15] Shen Z-K, Jackson DD, Ge BX (1996). Crustal deformation across and beyond the Los Angeles basin from geodetic measurements. J. Geophys. Res..

[16] Fujii Y (1979). Crustal Interaction between the south Kanto and the Tokai district, Japan: Latest crustal dynamics along the northern boundary of Philippine Sea Plate. Zisin.

[17] Ohtake M, Asada T (1983). Recent crustal movement in the Tokai area, Japan: As inferred from the seasonal effect corrected levelling data. Zisin.

[18] Kimata, F., Hirahara, K., Fujii, N., & Hirose, H. Repeated occurrence of slow slip events on the subducting plate interface in the Tokai region, Central Japan, the Focal Region of the Anticipated Tokai Earthquake (M = 8). American Geophysical Union, Fall Meeting 2001, abstract id. G31A-0126 (2001).

[19] Kobayashi A, Yoshida A (2004). Recurrence of the Tokai slow slip inferred from the tide gauge data at Maisaka. J. Geod. Soc. Jpn..

[20] Yamamoto E, Matsumura S, Ohkubo T (2005). A slow slip event in the Tokai area detected by tilt and seismic observation and its possible recurrence. Earth Planets Space.

[21] Okada Y (1992). Internal deformation due to shear and tensile faults in a half-space. Bull. Seismol. Soc. Am..

[22] DeMets C, Gordon RG, Argus DF (2010). Geologically current plate motions. Geophys. J. Int..

[23] Yokota Y, Ishikawa T, Watanabe S, Tashiro T, Asada A (2016). Seafloor geodetic constraints on interplate coupling on the Nankai Trough megathrust zone. Nature.

[24] Noda A, Saito T, Fukuyama E (2018). Slip-deficit rate distribution along the Nankai Trough, southeast Japan, with elastic lithosphere and viscoelastic asthenosphere. J. Geophys. Res. Solid Earth.

[25] Abe D, Yoshioka S (2022). Spatiotemporal distributions of interplate coupling in Tohoku, northeast Japan, for 14 years prior to the 2011 Tohoku-oki earthquake inverted from GNSS data. Tectonophysics.

[26] Akaike, H., Information Theory and an extension of the maximum likelihood principle. In *Second international symposium on information theory*. (eds B. N. Petrov, B. N. & Caski, F.) 267–281 (Akademiai Kiado, Budapest, 1973).

[27] Wessel P, Smith WHF (1998). New, improved version of the generic mapping tools released. EOS Trans. AGU.

[28] Bird P (2003). An updated digital model of plate boundaries. Geochem. Geophys. Geosyst..

