# Supplementary material for: Strain accumulation and release associated with the occurrence of SSEs in the subduction zones of the Japanese Islands
Source: Sci Rep. 2023 Jan 25;13:1379. doi: 10.1038/s41598-023-28016-1 (PMC9877015; doi:10.1038/s41598-023-28016-1)
Supplement: Supplementary file 1 — Supplementary Information. [file 41598_2023_28016_MOESM1_ESM.docx]

Supplementary Information for

**Strain accumulation and release associated with the occurrence of SSEs in the subduction zones of the Japanese Islands**

Hiroki Kawabata^1,*^ and Shoichi Yoshioka^2,1^

^1^ Department of Planetology, Graduate School of Science, Kobe University, Rokkodai-cho 1-1, Nada ward, Kobe 657-8501, Japan

^2^ Research Center for Urban Safety and Security, Kobe University, Rokkodai-cho 1-1, Nada ward, Kobe 657-8501, Japan

^*^corresponding author

e-mail: [h_kawabata@stu.kobe-u.ac.jp](mailto:h_kawabata@stu.kobe-u.ac.jp)

Contents

Text S1-S3

Table S1-S4

Figures and figure captions S1-S6

References

**Text S1 Results of the Tokai L-SSE**

The displacement, dilatation, maximum shear strain, and principal strain related to the Tokai L-SSE are shown in Fig. S3(a)-(d) for the period before the L-SSE, and in Fig. S3(e)-(h) during the L-SSE. Westward displacements can be dominant before the L-SSE, while the displacements turned to an east-southeast direction at the time of the L-SSE. In particular, the displacements in the northwestern part of Lake Hamana and from Lake Hamana to Omaezaki were large, with southeastward displacements up to approximately 10 cm.

The dilatation showed a large value of contraction just east of Lake Hamana before the L-SSE, but a large value of expansion at the same location during the L-SSE occurrence with a maximum value of$1.5\times{10}^{-6}$. This indicates that the dilatation shows significant expansion when the L-SSE occurred, where the contraction was large before L-SSE. In addition, contraction in the northwestern part of Lake Hamana was greater than that in its surrounding areas during the L-SSE.

For the maximum shear strain, negative values dominate before the L-SSE, while positive values are identified around Lake Hamana and Omaezaki during the L-SSE, indicating a temporal change from a compressive field to a tensile field. The strain was relatively large around Omaezaki before and during the L-SSE, with a maximum value of $1.9\times{10}^{-6}$ for the latter.

The principal strain field from Lake Hamana to Omaezaki shows northwest-southeastward compression before the L-SSE, which is consistent with the direction of subduction of the Philippine Sea plate, and changes temporally to north–south tension on the east side of Lake Hamana during the L-SSE. Comparing the amount of strain accumulation and release before and during L-SSE, respectively, the amount of strain release during the L-SSE was larger. However, as mentioned in the main text, the Tokai L-SSE cannot be traced back to the end of the previous Tokai L-SSE because of the problem on the starting date of the installation of GNSS stations. This lack of start date is the reason why the analysis period before the L-SSE is shorter.

Next, correlation coefficients were calculated for dilatation and maximum shear strain based on the amount of strain accumulated before the L-SSE and the amount of strain released during the L-SSE (Fig. S4(a), (b)). Fig. S2(a) shows 59 used calculation points with a displacement of 6.5 cm or greater at the time of the L-SSE. The correlation coefficients were -0.86 for dilatation and -0.35 for maximum shear strain, indicating a large negative correlation in the dilatation. These results suggest that the Tokai L-SSE is characterized by a large amount of strain release at the time of the L-SSE in the dilatation at locations where a large amount of strain accumulation prior to the L-SSE is identified.

**Text S2 Results of the Boso-Oki S-SSE**

The displacement, dilatation, maximum shear strain, and principal strain during the Boso-Oki S‒SSE for the period before the S‒SSE are shown in Fig. S5(a)-(d), and those during the SSE occurrence are shown in Fig. S5(e)-(h). The displacement shows that before the S‒SSE, the overall displacement field is oriented in the north‒northwest direction, and the amount of displacement increases towards the south of the Boso Peninsula. On the other hand, during the S‒SSE, displacement in the southeast direction was observed from the central to the eastern part of the Boso Peninsula. The displacement was larger towards the southeast, and the maximum displacement reached approximately 2.3 cm.

The dilatation showed contraction over the entire Boso Peninsula before the S‒SSE, but a region of slight expansion at the eastern edge of the central part of the Boso Peninsula was identified. One of the reasons for this dilation may be that the calculation points are located along the coast, and the GNSS stations used in the strain field calculations are biased towards the inland side. Such exceptional calculation points are eliminated in calculating the correlation coefficients. Although contraction was observed in the southern part of the Boso Peninsula at the time of the S‒SSE, almost the entire area showed expansion with a maximum value of $4.1\times{10}^{-7}$. In addition, the value of expansion was large in the region where the displacement was large at the time of the S‒SSE occurrence.

Comparing the period before the S‒SSE with that during the S‒SSE, we found that the maximum shear strain values also shifted from negative to positive for most of the region except for the southern part of the Boso Peninsula. Before the S‒SSE, by and large, the maximum shear strain was large from the central to the southern part of the Boso Peninsula, and during the S‒SSE, the maximum shear strain tended to increase towards the east in the central part of the Boso Peninsula. As is the case of dilatation, the regions with positive values exist in the eastern edge of the central part of the Boso Peninsula before the S‒SSE, and its reason may be the same as mentioned above.

Regarding the principal strain, the dominant direction before the S‒SSE was north–south compression, but during the S‒SSE, west‒northwest-east‒southeast tension became dominant. Comparing the amount of accumulated strain before the S‒SSE with the amount of released strain during the S‒SSE, the overall amount of released strain was smaller.

Correlation coefficients were also calculated for dilatation and maximum shear strain before and during the SSE (Fig. S6(a) and (b)). Fig. S2(b) shows the 21 calculation points whose displacement was 1.0 cm or greater during the S‒SSE. The correlation coefficients for dilatation and maximum shear strain were -0.74 and -0.66, respectively, showing negative correlations for both. However, for the maximum shear strain, the calculated points that shifted from contraction before the SSE to expansion during the S‒SSE were almost uniformly distributed in the region where the value of the maximum shear strain during the S‒SSE was more than $2.0\times{10}^{-7}$, so the negative correlation is considered to be weak. In the calculation points used to calculate the correlation coefficient, there is a location that shows negative values for both dilatation and maximum shear strain in the north of the S‒SSE occurrence region. This is because when the strain was released with the occurrence of the S‒SSE, the areas with relatively low strain accumulation contracted relative to the areas where large strain accumulation was identified before the S‒SSE occurred. From the above results, it was confirmed that the Boso-Oki S‒SSE tends to release the dilatation at the time of the SSE in the regions where the dilatation had been accumulated before the S‒SSE, indicating a strong relationship between the accumulation and the release in the dilatation.

**Text S3 The effects of correction of coseismic steps associated with the two large earthquakes on the results for the Bungo Channel L-SSE (2)**

For the Bungo Channel L-SSE (2), two large earthquakes, the 2011 Tohoku-Oki earthquake (11 March 2011, Mw 9.0) and the 2016 Kumamoto earthquake (16 April 2016, Mw 7.0), occurred during the strain accumulation period before the L-SSE (2), and the east–west component of the GNSS time series data shows coseismic steps caused by these earthquakes. The coseismic steps were not corrected in the analysis of the period prior to the SSEs in this study because we needed total accumulated displacements, including related effects, in the time series during the period before the L-SSE. Therefore, the accumulated years calculated by the method described in the main text may not provide an accurate estimation of the period prior to the SSEs. It is necessary to investigate what would have happened to the original Bungo Channel L-SSE (2) in terms of strain accumulation period and years if there had been no steps, i.e., strain perturbations, caused by these large earthquakes.

Therefore, we calculated the number of years of strain accumulation $t$ for the period before the L-SSE (2), correcting the coseismic steps caused by the two large earthquakes. More specifically, after correcting the steps when calculating the displacement prior to the L-SSE (2), we estimated the period prior to the L-SSE (2) for all stations such that the amount of displacement would be equivalent to the amount of accumulated strain prior to the L-SSE when the coseismic steps were not corrected. Then, the period before the L-SSE was determined when the steps were corrected by averaging the period before the L-SSE estimated at all stations. Thereafter, the number of years of strain accumulation was estimated by applying the same method.

As a result, the period before the L-SSE became 5.1 years, and the accumulated years were estimated to be 1.3 years. This value is smaller than the accumulation period described in the main text and is comparable to the accumulation period of the Bungo Channel L-SSE (1). Therefore, for the Bungo Channel L-SSE (2), the number of years of accumulated strain increased due to the 2011 Tohoku-Oki and 2016 Kumamoto earthquakes. In other words, the original period before the occurrence of the Bungo Channel L-SSE (2) is estimated to have been approximately 5.1 years. However, the accumulation of strain was delayed from the original period due to the release of some strain that had accumulated beneath the Bungo Channel by the occurrence of large earthquakes. As a result, the period before the occurrence of the Bungo Channel L-SSE (2) is considered to have increased. This indicates that some of the strain that had been accumulating before the Bungo Channel L-SSE (2) was released by the occurrences of both earthquakes.

**Table S1** Analysis period before and during the occurrence of each SSE.

| SSE | Number of observation stations | Analysis period prior to the SSE onset (years) | Analysis period during the SSE occurrence (years) |
| --- | --- | --- | --- |
| Tokai L-SSE | 97 | 1 January 1997 –  30 June 2000  （3.50） | 1 July 2000 –  30 June 2005  （5.00） |
| Boso-Oki S-SSE | 25 | 20 November 2002 –  8 August 2007  （4.72） | 9 August 2007 –  23 August 2007  （0.041） |
| Bungo Channel  L-SSE (1) | 95 | 7 August 2004 –  24 November 2009  （5.30） | 25 November 2009 –  6 February 2011  （1.20） |
| Bungo Channel  L-SSE (2) | 93 | 7 February 2011 –  19 April 2018  （7.20） | 20 April 2018 –  26 May 2019  （1.10） |

**Table S2** Threshold of displacement at the calculation point at each SSE.

| SSE (actual period) | Threshold (cm) |
| --- | --- |
| Tokai L-SSE  (1 July 2000 –  30 June 2005) | 6.5 |
| Boso-Oki S-SSE  (9 August 2007 –  23 August 2007) | 1.0 |
| Bungo Channel L-SSE (1)  (25 November 2009 –  6 February 2011) | 2.5 |
| Bungo Channel L-SSE (2)  (20 April 2018 –  26 May 2019) | 2.8 |

**Table S3** Correlation coefficient at each SSE.

| SSE (actual period) | Dilatation | Maximum shear strain |
| --- | --- | --- |
| Tokai L-SSE  (1 July 2000 –  30 June 2005) | -0.86 | -0.35 |
| Boso-Oki S-SSE  (9 August 2007 –  23 August 2007) | -0.74 | -0.66 |
| Bungo Channel L-SSE (1)  (25 November 2009 –  6 February 2011) | -0.82 | -0.20 |
| Bungo Channel L-SSE (2)  (20 April 2018 –  26 May 2019) | -0.84 | -0.59 |

**Table S4** Distance decay constant (DDC) at each SSE.

| SSE (actual period) | DDC (km) |
| --- | --- |
| Tokai L-SSE  (1 July 2000 –  30 June 2005) | 17.5 |
| Boso-Oki S-SSE  (9 August 2007 –  23 August 2007) | 17.5 |
| Bungo Channel L-SSE (1)  (25 November 2009 –  6 February 2011) | 20.0 |
| Bungo Channel L-SSE (2)  (20 April 2018 –  26 May 2019) | 20.0 |

(d)

(c)

(b)

(a)


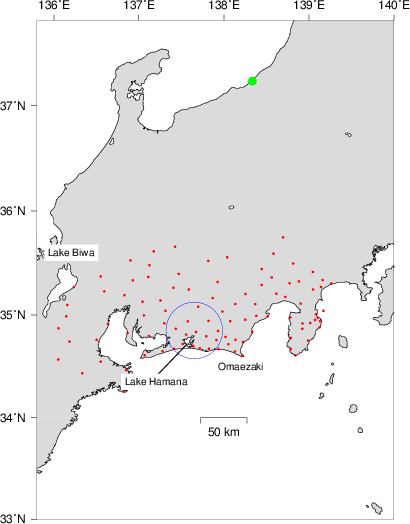

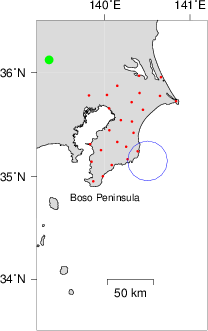

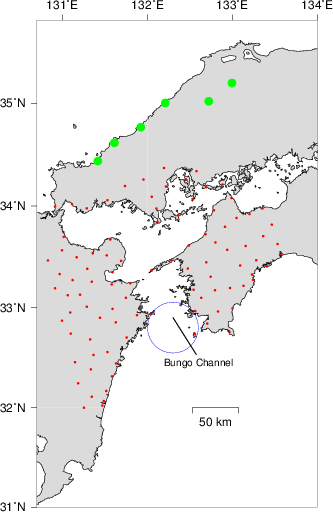

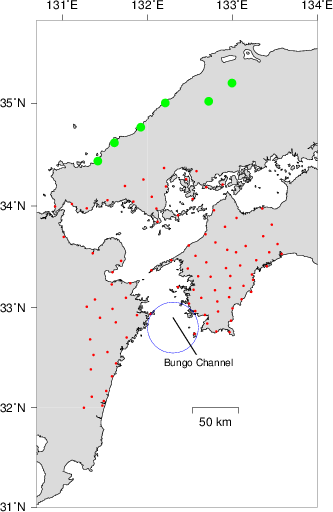


**Figure S1** Spatial distributions of GNSS stations used in the analysis before and during the occurrence of each SSE. Red and green dots represent the GNSS stations used in the analysis and reference stations, respectively, and blue encircled areas denote the approximate occurrence locations of each SSE. (a) Tokai L-SSE. Reference station: 950241. (b) Boso-Oki S-SSE. Reference station: 93005. (c) Bungo Channel L-SSE (1). Reference stations: 950407, 940076, 950388, 940075, 950385, and 950387. (d) Bungo Channel L-SSE (2). Reference stations: same as (c). The map was created by using the Generic Mapping Tools (GMT)^1^ (version: GMT3.4.6, URL link: <https://www.generic-mapping-tools.org/download/>)


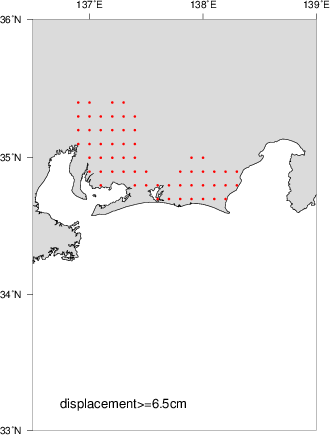

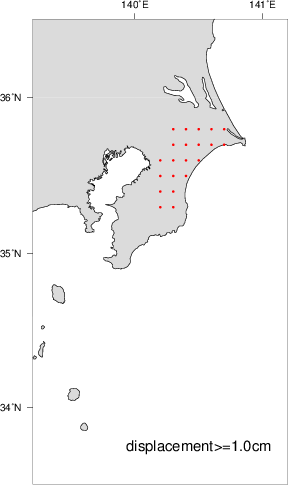

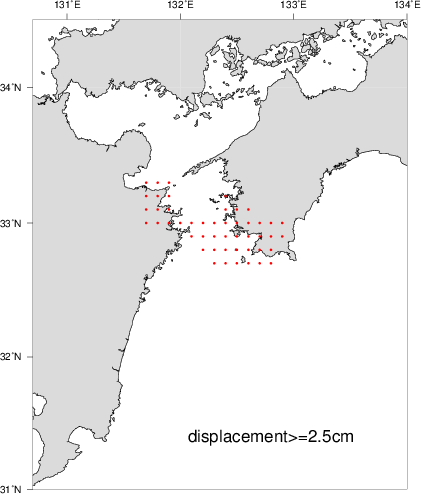

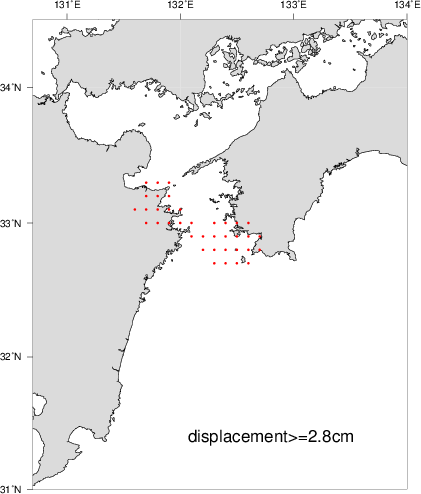


(a)

(b)

(c)

(d)

**Figure S2** Spatial distributions of the calculation points used to compute the correlation coefficients for each SSE. Red dots represent calculation points where the displacement exceeded a certain threshold value at the time of the SSE. (a) Tokai L-SSE. The threshold is 6.5 cm. (b) Boso-Oki S-SSE. The threshold is 0.5 cm. (c) Bungo Channel L-SSE (1). The threshold is 2.5 cm. (d) Bungo Channel L-SSE (2). The threshold is 2.8 cm. The map was created by using the Generic Mapping Tools (GMT)^1^ (version: GMT3.4.6, URL link: <https://www.generic-mapping-tools.org/download/>)


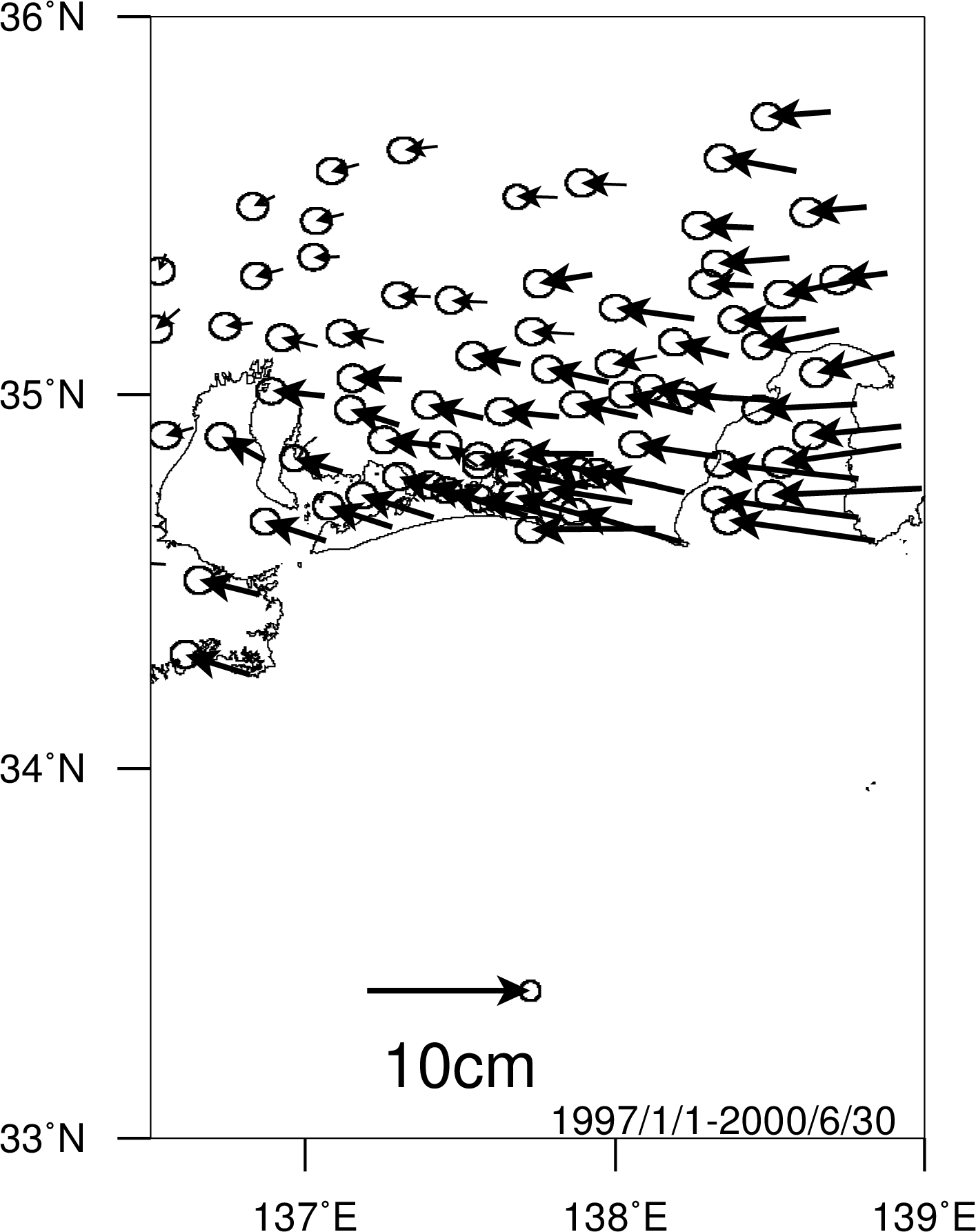

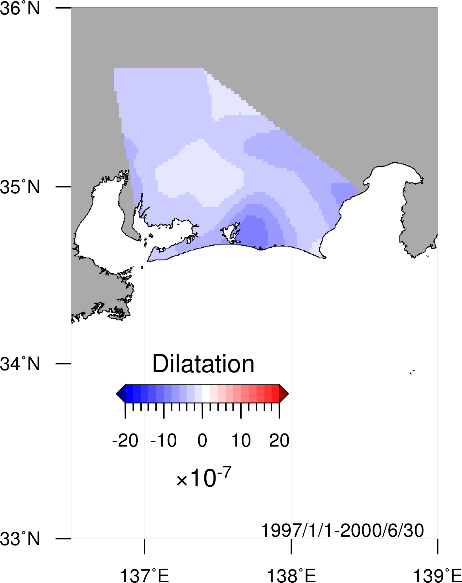

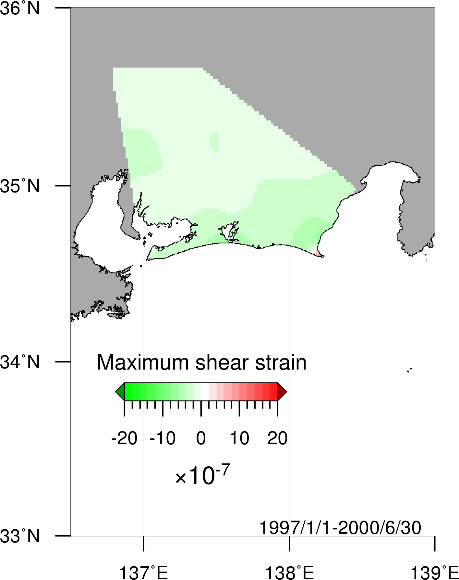

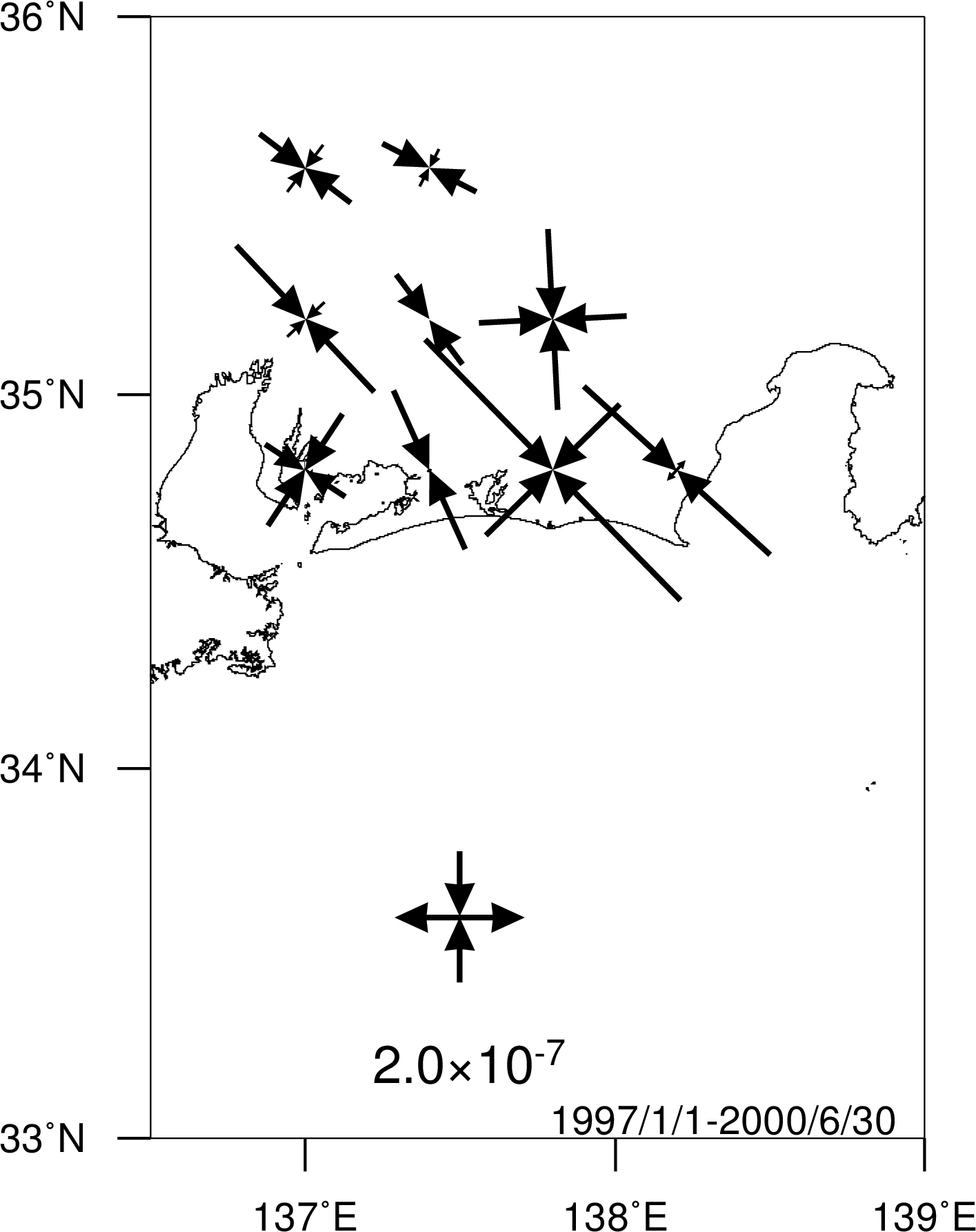


(a)

(b)

(c)

(d)


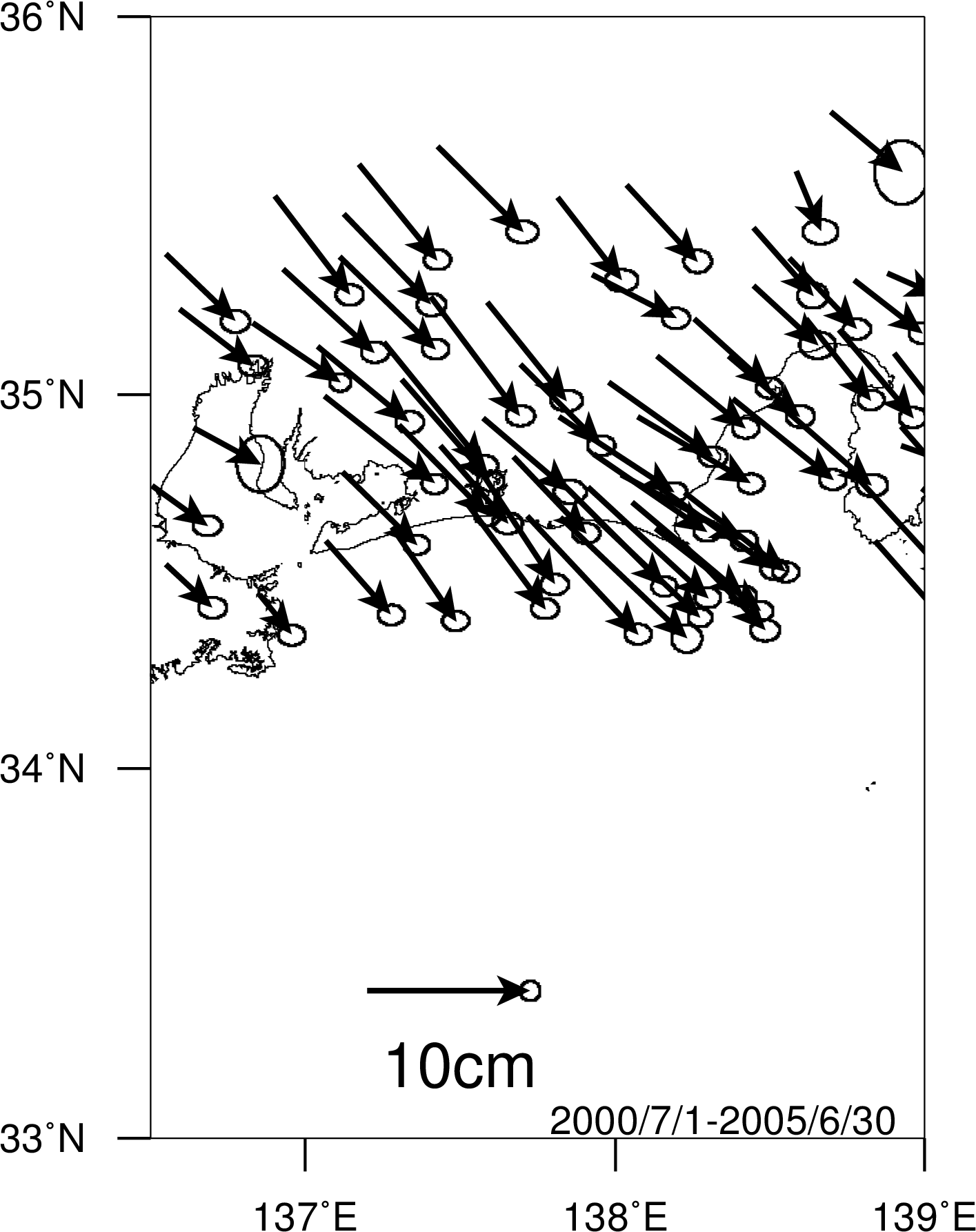

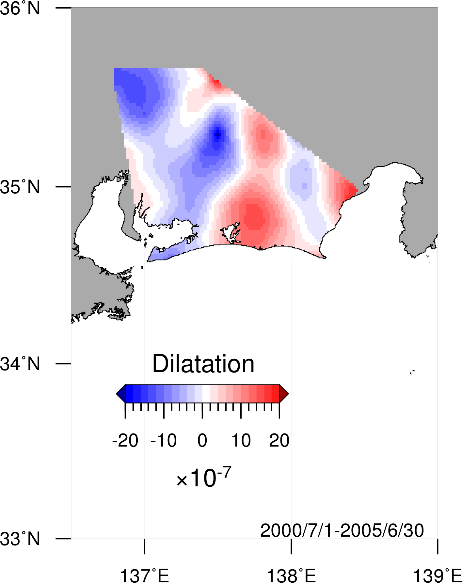

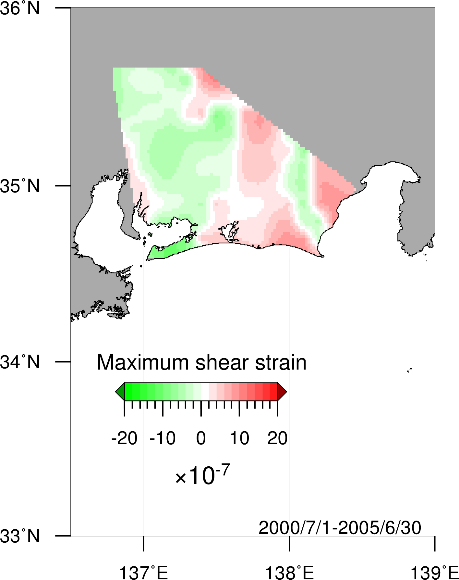

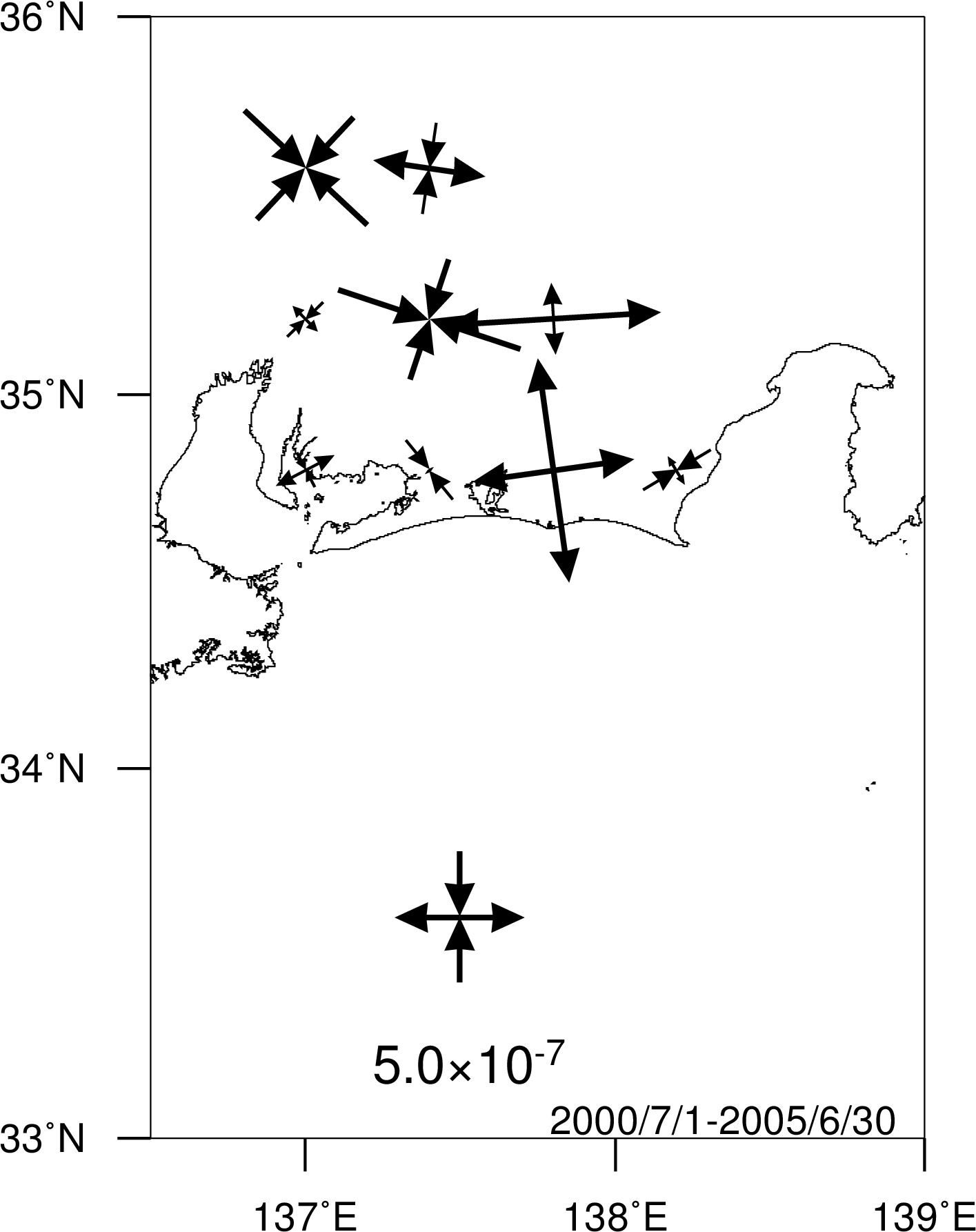


(e)

(f)

(g)

(h)

**Figure S3** Spatial distributions of displacement, dilatation, maximum shear strain, and principal strain before (1 January 1997-30 June 2000) and during (1 July 2000-30 June 2005) the Tokai L-SSE. The expressions of the figure are the same as those in Fig. 2(a)-(h).The map was created by using the Generic Mapping Tools (GMT)^1^ (version: GMT3.4.6, URL link: <https://www.generic-mapping-tools.org/download/>)

(a)

(b)


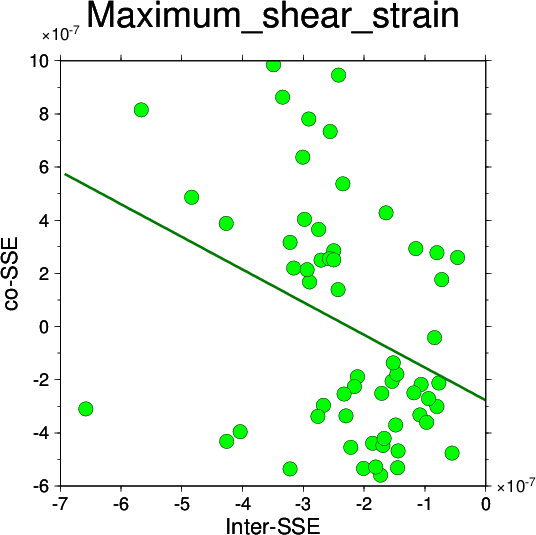

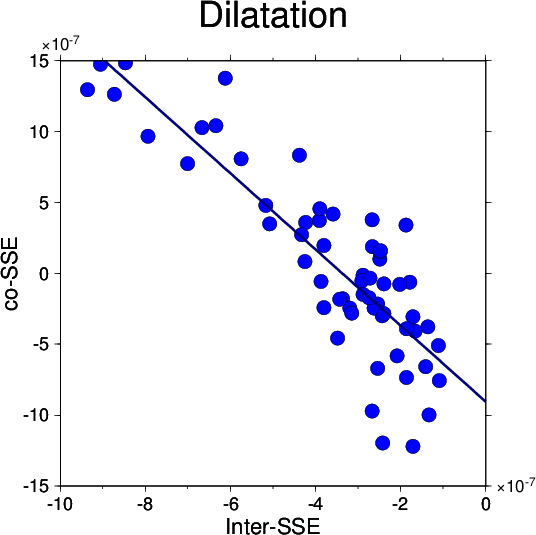


**Figure S4** Correlation diagram between the amount of accumulated strain before the Tokai L-SSE (1 January 1997-30 June 2000) and the amount of change in strain during the L-SSE occurrence (1 July 2000-30 June 2005). The values of the strain at the calculation points shown in Fig. S2(a) are used. The expressions of the figure are the same as those in Fig. 3(a) and (b).


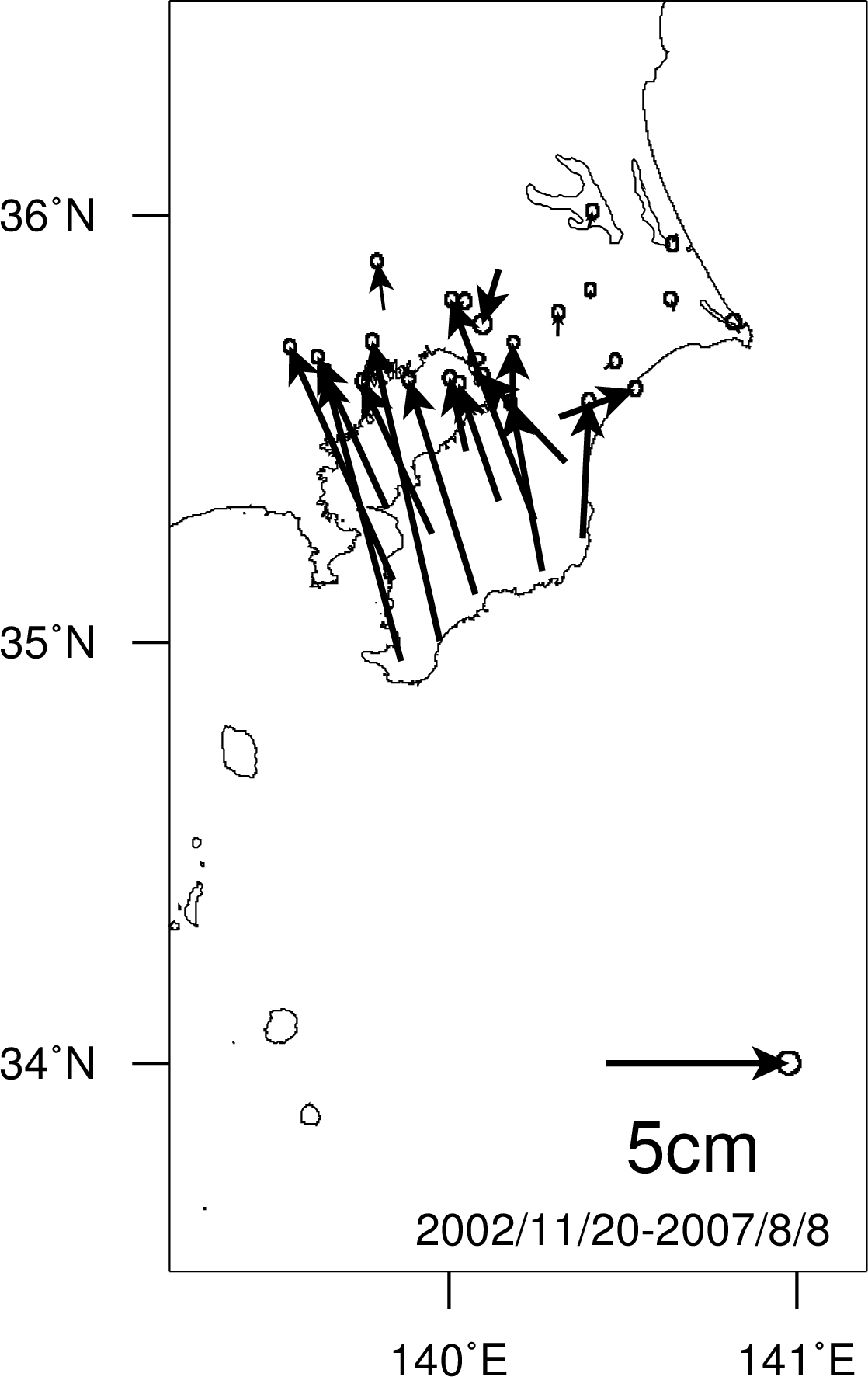

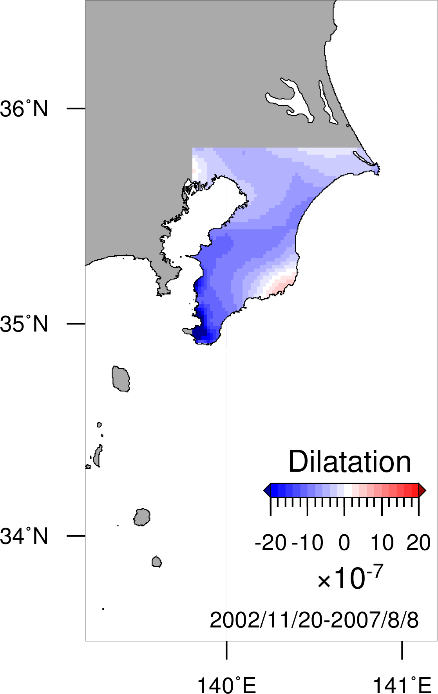

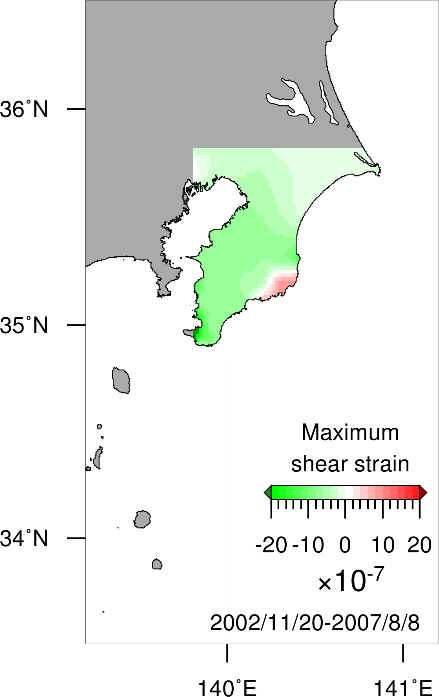

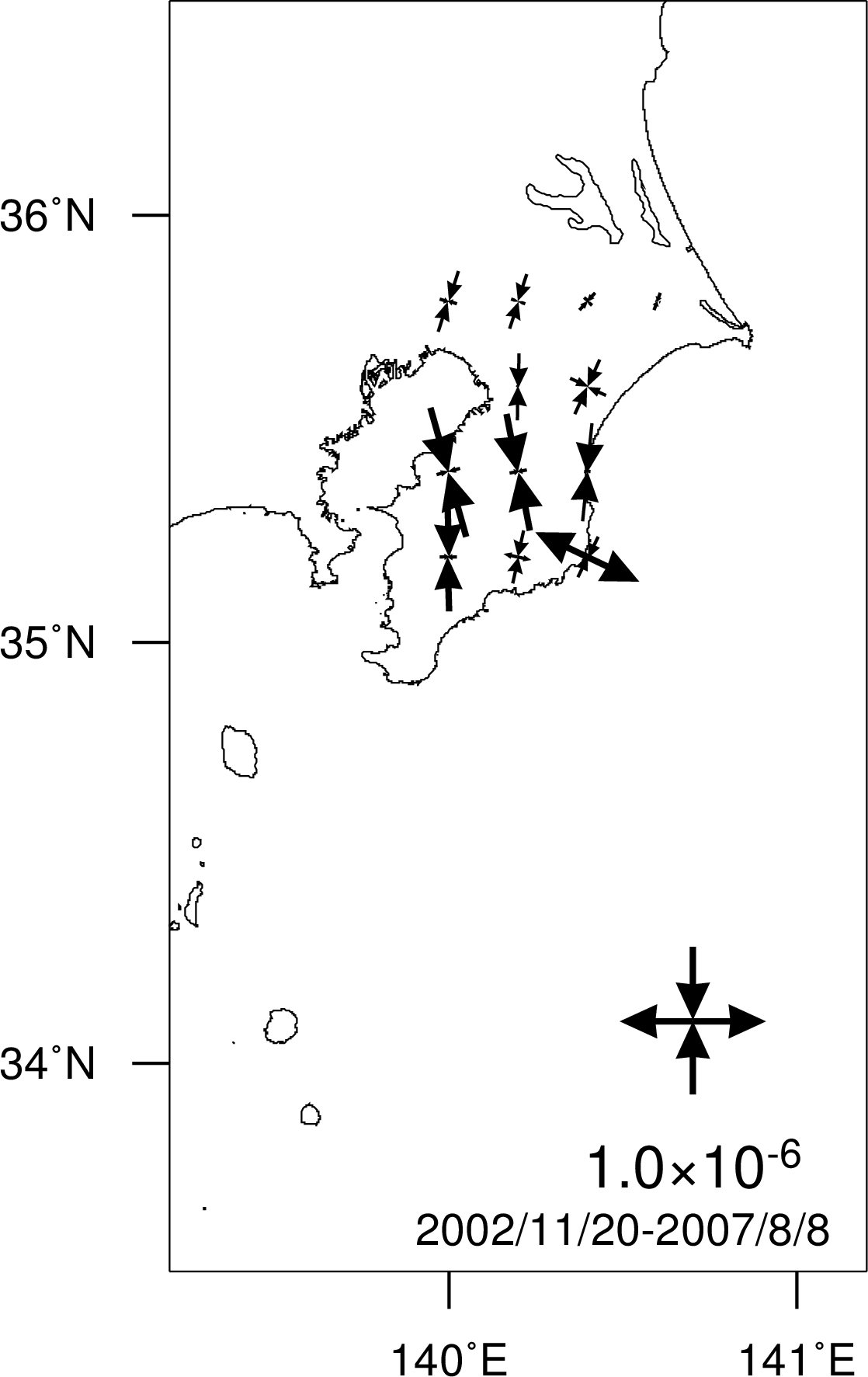


(a)

(b)

(c)

(d)


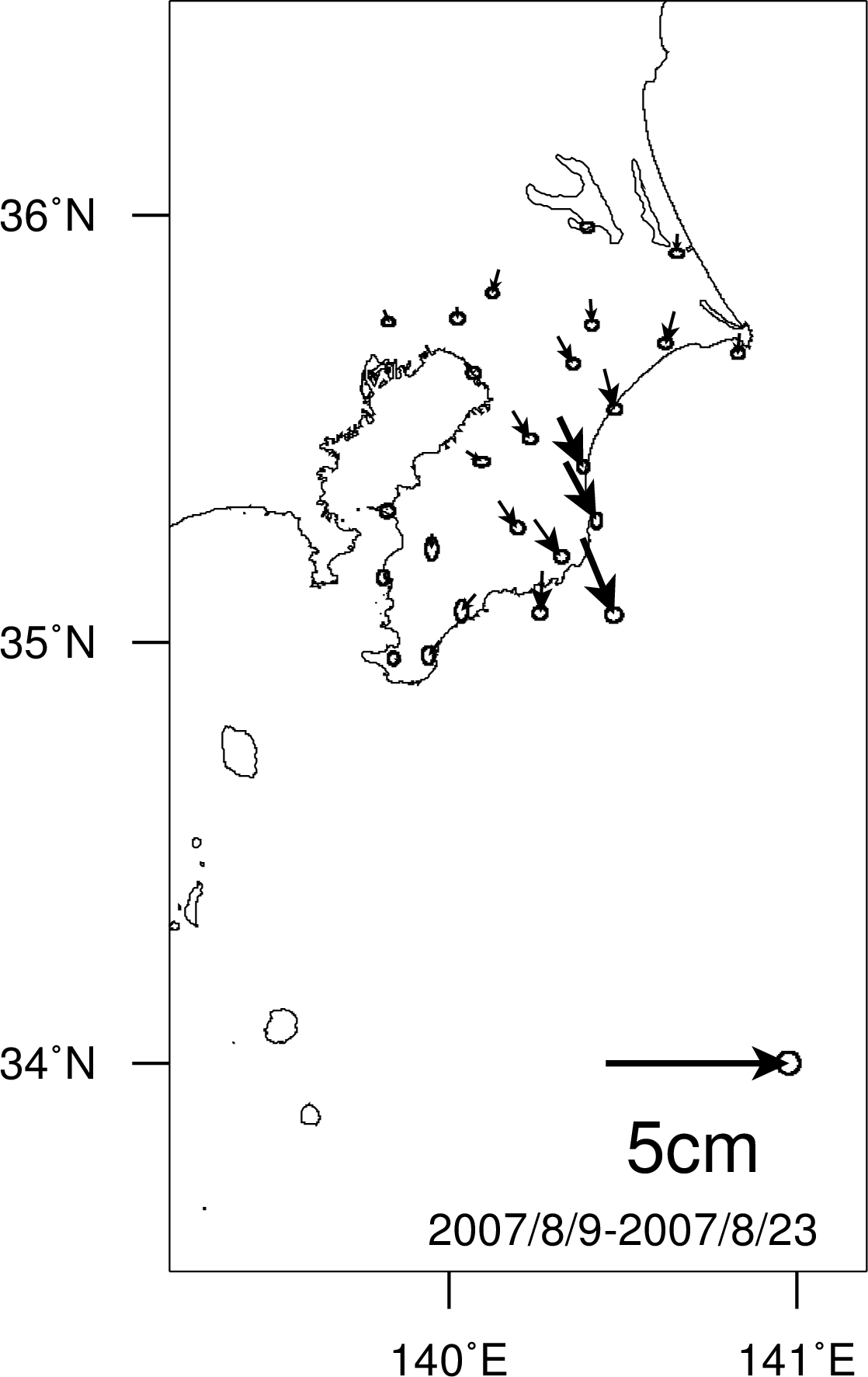

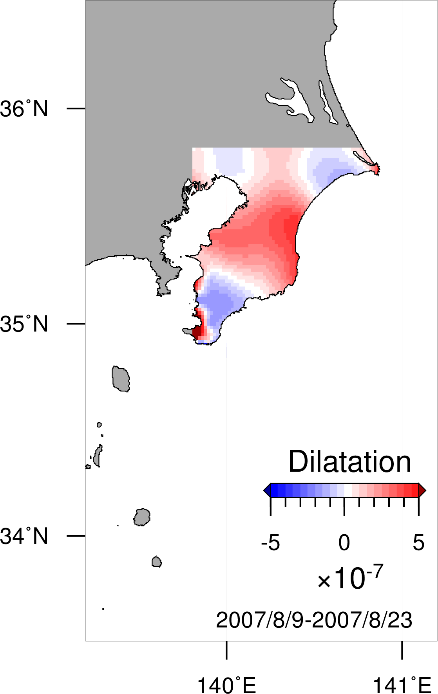

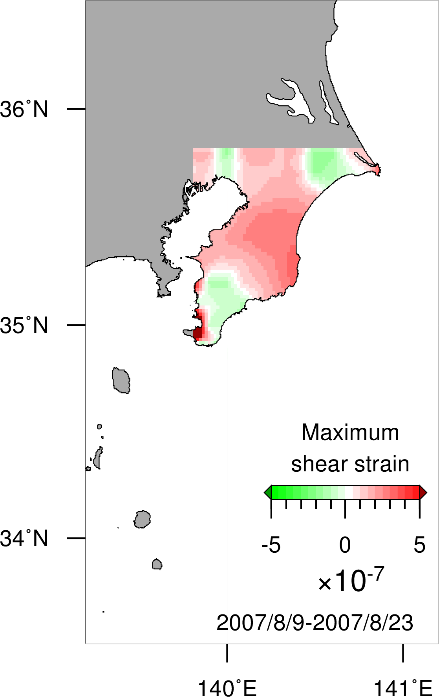

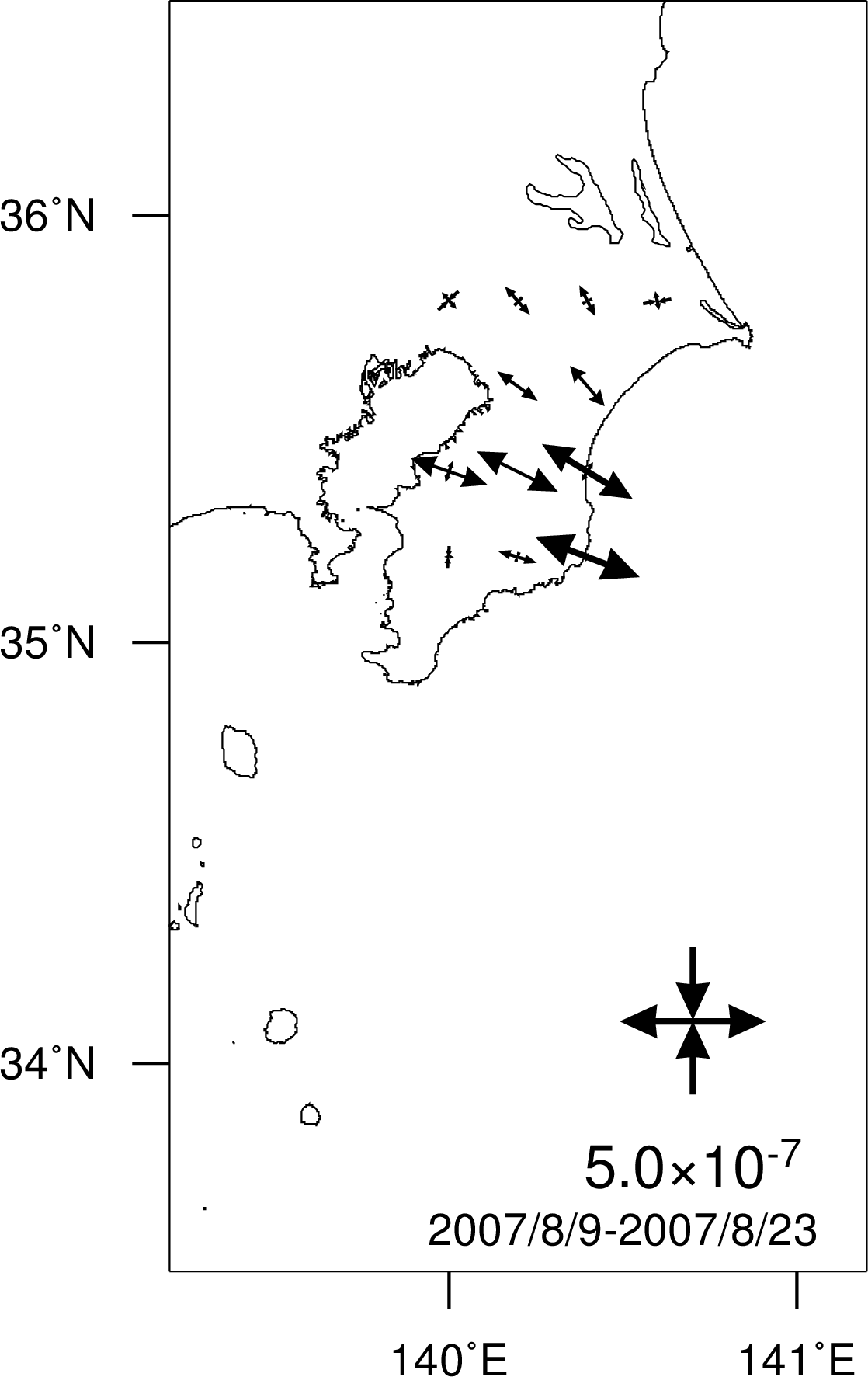


(e)

(f)

(g)

(h)

**Figure S5** Spatial distributions of displacement, dilatation, maximum shear strain, and principal strain before (20 November 2002-8 August 2007) and during (9 August 2007-23 August 2007) the Boso-Oki S-SSE. The expressions of the figure are the same as those in Fig. 2(a)-(h).The map was created by using the Generic Mapping Tools (GMT)^1^ (version: GMT3.4.6, URL link: <https://www.generic-mapping-tools.org/download/>)

(a)

(b)


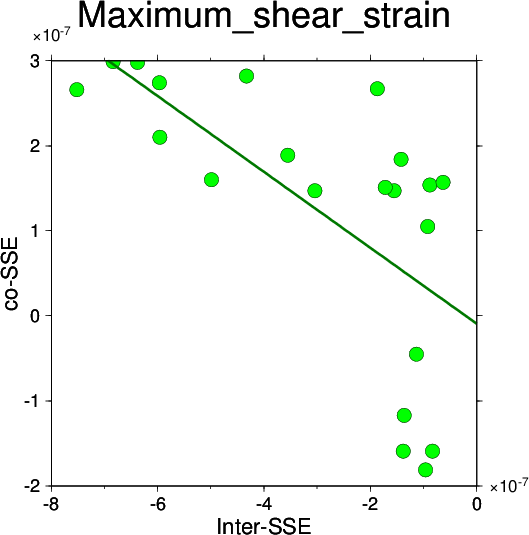

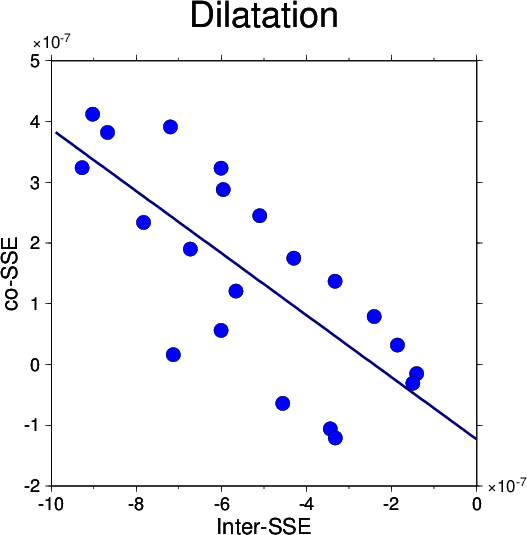


**Figure S6** Correlation diagram between the amount of accumulated strain before the Boso-Oki S-SSE (20 November 2002-8 August 2007) and the amount of change in strain during the S-SSE occurrence (9 August 2007-23 August 2007). The values of the strain at the calculation points shown in Fig. S2(a) are used. The expressions of the figure are the same as those in Fig. 3(a) and (b).

**References**

1. Wessel, P., and W. H. F. Smith, New, improved version of the generic mapping tools released. *EOS Trans. AGU* **79**, 579 (1998).
